# Detecting and Analyzing Politically-Themed Stocks Using Text Mining Techniques and Transfer Entropy—Focus on the Republic of Korea’s Case

**DOI:** 10.3390/e23060734

**Published:** 2021-06-09

**Authors:** Insu Choi, Woo Chang Kim

**Affiliations:** Department of Industrial and Systems Engineering, Korea Advanced Institute of Science and Technology (KAIST), 291 Daehak-ro, Yuseong-gu, Daejeon 34141, Korea; jl.cheivly@kaist.ac.kr

**Keywords:** politically-themed stock, sentiment analysis, transfer entropy, network analysis, investment strategy

## Abstract

Politically-themed stocks mainly refer to stocks that benefit from the policies of politicians. This study gave the empirical analysis of the politically-themed stocks in the Republic of Korea and constructed politically-themed stock networks based on the Republic of Korea’s politically-themed stocks, derived mainly from politicians. To select politically-themed stocks, we calculated the daily politician sentiment index (PSI), which means politicians’ daily reputation using politicians’ search volume data and sentiment analysis results from politician-related text data. Additionally, we selected politically-themed stock candidates from politician-related search volume data. To measure causal relationships, we adopted entropy-based measures. We determined politically-themed stocks based on causal relationships from the rates of change of the PSI to their abnormal returns. To illustrate causal relationships between politically-themed stocks, we constructed politically-themed stock networks based on causal relationships using entropy-based approaches. Moreover, we experimented using politically-themed stocks in real-world situations from the schematized networks, focusing on politically-themed stock networks’ dynamic changes. We verified that the investment strategy using the PSI and politically-themed stocks that we selected could benchmark the main stock market indices such as the KOSPI and KOSDAQ around political events.

## 1. Introduction

*Thematic stocks* generally refer to a group of stocks related to a particular topic and exist in various areas, including politics, science, technology, entertainment, environment, health care, and resource development. Thematic stocks in the same theme have the characteristic of synchronizing their stock price movements in the same direction. Investors invest in thematic stocks expecting high returns in the short term rather than stable returns from a long-term perspective. For this reason, thematic stocks’ price movements are generally decided by stock-related news or investors’ investing behaviors rather than by firms’ performance or financial status. In particular, stocks tied up with political themes are called *politically-themed stocks*, which are generally benefited from policies and politicians. By this definition, there can be two types of politically-themed stocks: *policy-related politically-themed stocks* and *politician-related politically-themed stocks*.

First, *policy-related politically-themed stocks* are defined as stocks that are expected to benefit from the policy direction of politicians or political parties. In the case of Joe Biden, the 46th U.S. president recently elected, environmental issues were cited as the most significant issue: he signed an executive order to rejoin the U.S. into the Paris climate agreement. As a result, companies in related industries such as solar, wind, and hydrogen power generation along with electric vehicles were expected to benefit as politically-themed stocks, since those industries can be supported by his eco-friendly policies. Likewise, industrial sectors related to specific policies are designated as policy-related politically-themed stocks.

Most studies of policy-related politically-themed stocks have validated their existence and effectiveness by measuring the *cumulative abnormal return* (CAR) of stocks during political events, especially during the presidential election. The term ‘*abnormal return*’ is the difference between the actual return of a security and the expected return, and CAR is the sum of all abnormal returns. The reason why prior researchers used CAR is to prove that they benefited during political events compared to the stock market. For example, Herron et al. [1] analyzed the link between the 1992 US presidential election and sector-by-sector returns in the US stock market. They argued that there were politically sensitive economic sectors in the 1992 presidential campaign period. Knight [2] reported significant changes in stock prices in the 2000 US presidential election by analyzing the policies of George W. Bush and Al Gore, both Republican and Democratic candidates, respectively. Levi and Yagil [3] provided evidence of correlations between the 2012 US presidential election result and the returns of stocks related to the policies of both Republican and Democratic candidates, Mitt Romney and Barack Obama, respectively. Gobran and Bacon [4] confirmed that the investment in policy-related stocks before the presidential election could generate supra-normal returns. Wagner, Zeckhauser, and Ziegler [5] demonstrated the company stock price reactions to the 2016 election from corporate tax policies.

*Politician-related politically-themed stocks* are defined as stocks that are privately related to major stakeholders related to politicians. At this time, it means that the major stakeholders of a particular company have a form of relationship that has no relation with the politicians’ policy direction. Instead of their policy direction, those stakeholders are connected to politicians based on blood ties, regional ties, academic ties, etc. These politician-related politically-themed stocks occur very frequently in the Republic of Korea (hereafter referred to as *Korea*). This is because, unlike other countries, the concept of politician-related politically-themed stocks is more widely accepted in the Korean stock market. For this reason, most research of politically-themed stocks in Korea is based on politician-related politically-themed stocks. The Financial Services Commission and Financial Supervisory Service in Korea warned of the expected danger of investing in politically-themed stocks. They claimed that politically-themed stocks related to presidential candidates led to abnormal returns before the nineteenth presidential election in Korea but rapidly plunged after the election [6,7]. Nam [8] investigated politically-themed stocks that showed unusual price spikes during the sixteenth to nineteenth presidential election periods. He demonstrated that the results of presidential election periods affected the politically-themed stocks of winners and losers differently and alerted that investors need their attention in investing those stocks. Kwak and Yeo [9] documented that the significant short-term and long-term positive CAR of politically-themed stocks related to some presidential candidates occurred during the nineteenth presidential election in Korea.

In sum, most empirical studies of politically-themed stocks argued that politically-themed stocks generate positive CAR during political event periods, regardless of the types of politically-themed stocks [1,2,3,4,5,6,7,8,9]. However, no verification has been made on causal evidence of whether the occurrence of abnormal returns is actually affected by policy moves or politicians.

In this research, we focused on the politician-related politically-themed stocks, not on the ones benefiting from the politicians’ policies, but on the ones benefiting from politicians’ connections. (From here, the politically-themed stocks that we will discuss means politician-related politically-themed stocks.) These politically-themed stocks are mainly determined very subjectively and can be spanned among multiple industrial sectors since private connections between politicians and those stocks’ major stakeholders are irrelevant to industrial sectors. Thus, the methodology for classifying politically-themed stocks was not presented explicitly in existing studies and was determined by the opinions of prior researchers.

We have set the scope of our research to Korea because these politically-themed stocks occur more frequently than in other countries and are strongly influenced by politicians’ reputations. The phenomenon of politicians’ reputations affecting the stock price can be interpreted as the result of frequent business–government collusion in Korea [10,11,12,13]. For example, negative events such as legal punishment could diminish politicians’ political influence and cause negative public opinions towards them. These results can lead to a decline in politically-themed stocks [6,7,8,9].

According to existing research, these politician-related politically-themed stocks have several characteristics, which can be summarized into two representative features. The first is the concept of ‘leading stocks’. In Korea, if the connection between a politically-themed stock’s stakeholder and a politician is deeper than other stakeholders, the stock is generally called the leading stock. For example, if a stakeholder of a politically-themed stock is a politician’s family member, this stock is usually classified as the leading stock of the politician’s politically-themed stocks. These stocks are classified as the core of the particular politician’s politically-themed stocks and served as a signal of other politically-themed price movements. These leading stocks are frequently used in Korea when financial regulatory authorities look for signals of politically-themed stocks’ abnormal movements. The second characteristic is the rapid stock price fluctuations that occur during political events, such as election periods, and the positive CAR derived from those price fluctuations. This positive CAR effect, known primarily for politicians who have achieved good results during political events, stems from the expectation that future politicians’ actions will play a positive role in stock returns [6,7,8,9].

Due to these characteristics of politically-themed stocks, politically-themed stocks in Korea often have abnormal trading phenomena: occurring sharp rises and falls, or increasing turnover rates depending on the actions of these politicians. Furthermore, whether stakeholders are related to influential politicians is highly unclear, thus most of that information relies on rumors. For these reasons, politically-themed stocks are sometimes closely linked to financial crimes such as market manipulation, spreading false information, etc. Therefore, financial regulatory authorities in Korea judged that overheated investments in politically-themed stocks could cause enormous losses to individual investors and add to market inefficiency by increasing the number of unfair trades. This was the reason why they tried to crack down on, regulate, and audit the companies classified as politically-themed stocks for the last 5 years [6,7,8,9].

Additionally, stocks designated as politically-themed stocks in these studies were qualitatively and subjectively set by researchers, and no quantitative verifications were made. Studies related to politically-themed stocks presumed all expected stocks that were known to be linked to certain politicians, such as blood ties, regional ties, academic ties, etc. [6,7,8,9]. However, some of them did not have a clear positive CAR effect and moved very differently compared to other politically-themed stocks [9].

This study attempted to select politically-themed stocks as information flows from the positive or negative reputation of politicians to abnormal returns in order to supplement the methodologies of existing research. In the process, politically-themed stocks were selected as stocks with statistically significant causal relationships from the positive or negative reputation of politicians. We then used selected politically-themed stocks to validate the two characteristics: the presence of leading stocks and the occurrence of positive CAR effects around political events.

To validate this idea, the recent 5 years (2016–2020) were set as research periods. The 12 politicians with experience in presidential elections or politicians who had been mentioned as strong candidates in presidential elections were selected in research periods [1,2,3,4,5,6,7,8,9]. Then, we calculated the politician sentiment index (PSI) to quantify public opinions about those politicians. We collected politicians’ search volume data and text data related to politicians such as news, articles, editorials, and comments from two search engines, Naver and Google, since their search shares in Korea accounted for over 85% within the research period. Then, we analyzed the collected text data using sentiment analysis techniques and combined the search volume data and sentiment analysis results to calculate PSI. Then, we chose politically-themed stock candidates considering the politician-related search volume data and search results. After the above process, we verified causal relationships from ROC of the PSI to abnormal returns of politically-themed stock candidates. If they have statistically significant causal relationships, we concluded that they were affected by the politician and selected them as politically-themed stocks. Using selected politically-themed stocks, we verified the representative characteristics of politically-themed stocks in Korea through causal relationships, and established networks of politicians’ politically-themed stocks (hereinafter referred to as the political-themed stock networks) based on their causal relationships. From political-themed stock networks, we verified the existence of leading stocks based on the size of causal relationship measures. We then constructed and validated an investment strategy using politically-themed stocks to verify the prior research results of politically-themed stocks: CAR effects occur around political events [1,2,3,4,5,6,7,8,9]. Especially, the investment strategy is based on tracking the positive public reputation of selected politicians. This is due to politically-themed stocks’ CAR occurring more positively in Korea if the politician obtains benefits from political events, such as winning the election [6,7,8,9].

The remainder of this paper is organized as follows. In Section 2, we explain the data used in this research and conduct preliminary analyses. Then, we present methodologies to select politically-themed stocks based on the results of preliminary analyses. Section 3 delineates the selection processes and results of politicians’ politically-themed stocks. Then, we construct politicians’ politically-themed stock networks using selected politically-themed stocks and analyze the known characteristics of politically-themed stocks. Section 4 discusses the main results of this paper. Lastly, Section 5 provides the concluding remarks.

## 2. Data and Methodology

### 2.1. Research Period

In this study, the research period was set from 1 January 2016, to 30 June 2020. We selected this period because all three primary elections in Korea were held at intervals of a year, and unique political situations (impeachment of the Eighteenth President) were included in this research period. The five major political events that occurred during the period in Korea are in Table 1.

### 2.2. Data

#### 2.2.1. Politician-Related Data

We used web search data and included search volumes and associated search keywords in this study. First of all, we selected 12 politicians in Korea for our research subjects. These selected 12 politicians with experience in presidential elections or politicians who had been mentioned as strong candidates in presidential elections were selected in the research periods [1,2,3,4,5,6,7,8,9]. Daily search volume data of politicians in the research period were originated from Naver Trends and Google Trends. As we mentioned before, we selected those two search engines because their search shares in Korea occupied over 85% within the research period. All 12 chosen politicians were de-identified using letters A to L randomly because this paper’s purpose was not to comment on a particular politician or to encourage the sale of stocks for a specific company. The brief information of 12 selected politicians are in Table 2.

**Figure 1:**
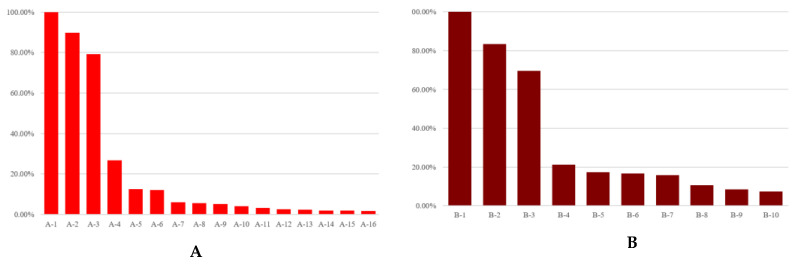

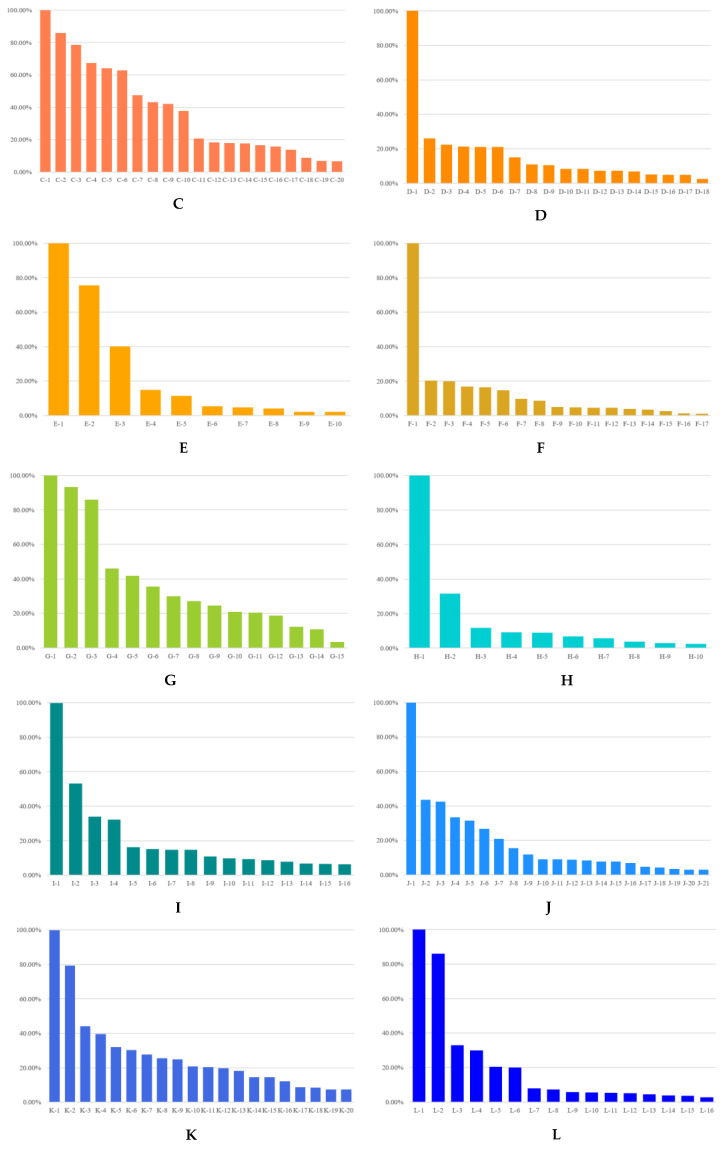
Politically-themed stock candidates and their politician-related search volumes. Note: The characters of subfigures (**A**–**L**) mean the politicians’ assigned alphabet for de-identification.

We noted that the larger the theme-related search volume data, the more these thematic stocks are usually known as leading stocks. Therefore, we searched through keywords such as ‘politically-themed stocks of politician A’ or ‘politician A’s politically-themed stocks’. Using collected search volume results based on those keywords, we designated the politician-related stocks whose search volumes were more than 1% of the search volume of most searched politician-related stocks as the politically-themed stock candidates from the search results. For example, if the search volume of the most searched politician-related stocks is 200,000, then the politician-related stocks whose search volumes are over 2000 are designated as the politically-themed stock candidates. As a result, we obtained 189 politically-themed stock candidates. A range of 10 to 21 stocks was allocated as political-themed stock candidates for 12 politicians each. As mentioned before, 189 politically-themed stock candidates were de-identified, just as politicians. According to the politician-related search volumes, they were numbered in descending order—for example, the stock of the seventh most searched politically-themed stock of politician A is labeled A-7. Using de-identified numbers, we observed whether the actual quantity of causal relationships or network measures are related to the politician-related search volumes. Figure 1 summarizes the number of politically-themed stock candidates for each of the politicians and the ratio of each stock’s politician-related search volume to the most searched stock’s politician-related search volumes.

#### 2.2.2. Text Data

We randomly collected another 35,019 articles from the political section of Naver News and Google News from 2015 to 2020 and labeled them with sentiment lexicons. We labeled sentiment values of those articles 1 if the summation of lexicons in an article is positive and −1 if the summation of lexicons in an article is negative. We assumed that each article has only one sentiment in one article: positive or negative. After labeling those data, we used them as training data for conducting sentiment analysis. Additionally, we collected 216,843 text data related to 12 politicians in training days of research periods from the political section of Naver News and Google News. To collect the politician-related text data, we collected the text data including the politicians’ names. Using the trained model, we conducted sentiment analysis using these collected text data for calculating the daily sentiment score of 12 politicians.

#### 2.2.3. Abnormal Return Data

In this study, we verified politically-themed stocks using the methods that try to calculate politicians’ positive and negative reputation quantitatively in the form of PSI and checked causal relationships to abnormal returns of their politically-themed stocks. Accordingly, articles, editorials, and comments related to politicians were selected as text data for performing sentiment analysis to avoid strong biases of unfiltered documents in private spaces such as social network services and blogs. Then, we performed sentiment analysis of those collected text data and calculated the PSI for politicians within the research period. Using the calculated PSI, we verified causal relationships from the ROC of the PSI to abnormal returns of politically-themed stock candidates. Then, the stocks with statistically significant causal relationships were selected as politically-themed stocks.

In this study, we used abnormal returns of stocks to reduce a stock market’s influence and focus on politically-themed stocks’ performances on the individual level. The abnormal return of an asset is the subtraction of the expected return derived from the asset pricing model from the historical return. To calculate the abnormal returns of stocks, we collected the closing price data of 189 politically theme stock candidates in the research period and computed abnormal returns using the market model (MM).

MM was introduced by Brown and Warner [14] to examine the properties of daily stock returns and how particular characteristics of these data affect event study methodologies. Various finance studies have used this model to derive individual stocks’ abnormal returns [9,15]. The equation is as follows:

(1) $$E(r_{i,t})=\alpha_{i}+\beta_{i}E\left(r_{m,t}\right)+\varepsilon_{i,t}.$$

In Equation (1), $E(r_{i,t\;})$ is the return of stock i on day t and $E(r_{m,t})$ is the expected daily return to the market portfolio of risky assets on day t. $\alpha_{i}$ and $\beta_{i}$ are the intercept and the slope of the fitted line derived from linear regression results. We estimated the values of $\alpha_{i}$ and $\beta_{i}$ using ordinary least squares (OLS) estimators in the original research. Therefore, $\varepsilon_{i,t}\;$is the error term (a random variable) with expectation zero and finite variance. Additionally, $\varepsilon_{i,t}\;$is uncorrelated to the market return $E\left(r_{m,t}\right)$ and firm return $E(r_{i,t})$ with i≠j, homoskedastic and not autocorrelated. The daily returns of Korean Securities Dealers Automated Quotations (KOSDAQ) and Korea Composite Stock Price Index (KOSPI) indices are used as $E(r_{m})$ in this study. As a result, we can derive abnormal returns with estimated coefficients $\hat{\alpha_{i}}$ and $\hat{\beta_{i}}$ from Equation (2). In Equation (2), $r_{i,t}$ is the actual return of stock i on day t and $r_{m,t}$ is the actual return of the market index on day t.

(2) $${\mathrm{AR}}_{i,t}=r_{i,t}-\left[\hat{\alpha_{i}}+\hat{\beta_{i}}r_{m,t}\right]$$

#### 2.2.4. Preliminary Analysis

We used the abnormal return dataset derived from the market model for measuring causal relationships from ROC of the PSI to abnormal returns of politically-themed stocks. This dataset consists of 1101 daily abnormal return data of 189 politically-themed stock candidates. We checked elementary statistics and performed the Shapiro–Wilk test and Jarque–Bera test for normality, the Ljung–Box test for 10-order serial autocorrelation in squared returns. Additionally, the augmented Dickey–Fuller (ADF) test, Phillips and Perron (PP) test, and Kwiatkowski–Phillips–Schmidt–Shin (KPSS) test for stationarity and the White test for homoskedasticity. The detailed results of abnormal return data are in Table A1 of Appendix A.

**Figure 2:**
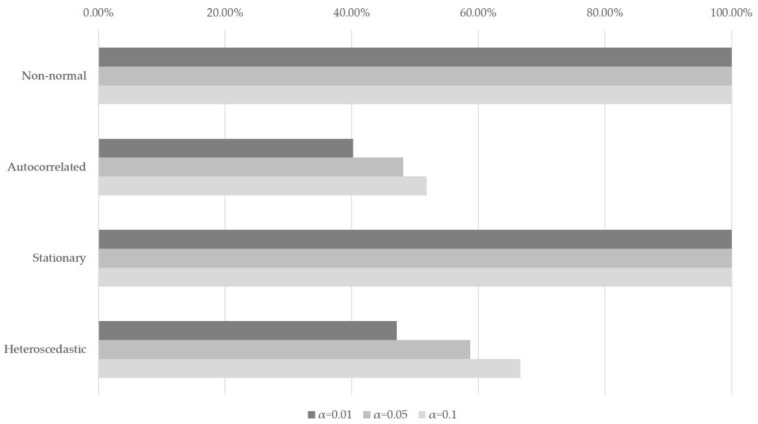
Summary of test results of abnormal returns of 189 stocks.

We summarized our diagnostic results in Figure 2. Figure 2 shows that our abnormal return data exhibit departure from normality based on the Shapiro–Wilk test and Jarque–Bera test. The two test results show that our data do not meet normality regardless of sample sizes at all three significance levels at the rate 0.1, 0.05, and 0.1. Additionally, all abnormal return data also turned out stationary according to ADF, PP, and KPSS tests at all three significance levels. We chose the ADF test results as the representative of those three stationary tests for brevity. In Figure 2, the bar charts mean that the number of stocks that are satisfied with the statistical properties of data that are written at the left side at the significance level 0.1, 0.05, and 0.01 each.

### 2.3. Methodology

#### 2.3.1. Sentiment Analysis

Sentiment analysis is a text-mining methodology that analyzes the attitude or inclination of writing or talking to identify sentiments on a particular subject, usually the text data. It is mainly used to determine the positive and negative opinions of texts such as articles, movie reviews, or posts from social network services.

From the aspect of tasks and applications, sentiment analysis has been applied in broad areas such as subjectivity classification, polarity determination, multilingual and cross-lingual sentiment analysis, cross-domain sentiment analysis, opinion spam detection, corpora creation, opinion word, aspects extraction, etc. Concerning the finance domain, researchers have used sentiment analysis to predict the movements of stock prices, support decisions, and anticipate risks.

We assumed that sentiments within one document extracted from articles, editorials, comments, and posts, were probability-distributed to calculate the PSI using the sentiment analysis. We used a machine learning-based approach for performing sentiment analysis implementation: the long short-term memory (LSTM) [16] model. LSTM is one of the recurrent neural network (RNN) techniques that are well-suited to time-series data, presented by Hochreiter and Schmidhuber, and has been usually used in language-based machine learning and speech recognition. LSTM can be used to categorize or predict future data with macroscopic attention to historical data and has been used as one of the most commonly used machine learning models to conduct sentiment analysis of Korean texts in recent years [17,18]. Especially, the bidirectional LSTM (BiLSTM) model is used in this study because BiLSTM is known as the proper model for sentiment analysis using the Korean language [19,20].

LSTM generates positive rates and negative rates of documents. Using the sentiment analysis results, the PSI of politicians can be defined as in Equation (3) so that the search volume data and sentiment analysis results can be considered simultaneously.

(3) $${\mathrm{PSI}}_{i,\;t}=\frac{1}{2}\left(\overset{V_{t}}{\underset{j=1}{\sum }}\frac{P_{i,\;t}^{j}-N_{i,\;t}^{j}}{P_{i,\;t}^{j}+N_{i,\;t}^{j}}+1\right)\frac{{\mathrm{SV}}_{i,\;t}}{V_{i,\;t}}=\left(\overset{V_{t}}{\underset{j=1}{\sum }}P_{i,\;t}^{j}\right)\frac{{\mathrm{SV}}_{i,\;t}}{V_{i,\;t}}$$

In Equation (3), ${\mathrm{PSI}}_{i,\;t}$ is the PSI of the politician i at time t, $P_{i,\;t}^{j}$ is the positive rate and $N_{i,\;t}^{j}$ is the negative rate of the j-th document of politician i at time t from sentiment analysis results which hold $P_{i,\;t}^{j}+N_{i,\;t}^{j}=1$. $V_{i,\;t}$ means the number of documents related to politician i used in analysis at time t, and ${\mathrm{SV}}_{i,\;t}$ is the T-score of search volume data of politician i at time t. We converted search volume data to T-score because their rate of changes (ROC) are too extreme to use. We also transformed original related terms of $P_{i,\;t}^{j}$ and $N_{i,\;t}^{j}\;$from scale [−1, 1] to [0, 1] for the convenience of calculation. We obtained the daily PSIs of politicians by calculating (3) and computed their rate of changes to demonstrate causality between the abnormal returns of politically-themed stock candidates. Using the property that $P_{i,\;t}^{j}+N_{i,\;t}^{j}=1$, we can convert the PSI to the rightmost expression of (3). We obtained the daily PSI of politicians by calculating (3) and computed their ROC to demonstrate causal relationships between abnormal returns of politically-themed stock candidates.

#### 2.3.2. Transfer Entropy

To select and analyze politically-themed stocks, we needed to measure causal relationships quantitatively. However, general causal relationships represented by Granger causality [21] required some assumptions of data like normality, stationary, and linearity. However, the results of the preliminary analysis show that the abnormal returns of politically-themed stock candidates were not satisfied with normality. This is similar to the known natures of return-based data of stocks, in that they do not usually satisfy these properties [22,23,24]. Therefore, we tried to utilize econophysics and information theories, which can be used without the assumptions mentioned above. From those theories, we can consider not only linear relationships but also non-linear relationships between objectives to measure causal relationships [25,26]. Accordingly, we used the concept of transfer entropy (TE) proposed by Schreiber [27], which are the entropy-based measures. In detail, we used effective transfer entropy (ETE) using Shannon entropy in this study.

TE is a non-parametric measure for verifying the information transfer amount between two variables based on Shannon entropy. Due to its feature to quantify causal relationships within systems and to identify source variables and target variables efficiently, TE has been spotlighted and widely used not only in the information or physics field but also in neuroscience, electrical engineering, chemical engineering. TE has been widely used to determine causal relationships between financial assets and markets in finance [28]. Specifically, general stock market indices, exchange rates, stock price, sector index, and cryptocurrency has been researched using TE. In the 2000s, Marschinki and Kantz [29] reported the causal relationship between the German DAX Xetra Stock Index (DAX) and Dow Jones Industrial Average. Kwon and Yang [30] showed the directionality of the information transfer and found that the market indices influence individual stocks in the US stock market. After the 2000s, Dimpfl and Peter [31] analyzed the causal relationship of the credit default swap market relative to the corporate bond market for the pricing of credit risk and the dynamic relation between market risk and credit risk proxied by the VIX and the iTraxx Europe from the perspective of pre-crisis, crisis and post-crisis periods based on ETE.

Moreover, Sandoval [32] used ETE to examine the causal relationship among 197 worldwide financial companies. Sensoy et al. [33] investigated the strength and direction of information flow between exchange rates and stock prices in several emerging countries using ETE. Based on TE, Bekiros et al. [34] investigated the network dynamics in US equity and commodity markets, and Lim et al. [35] analyzed the information flow between industrial sectors in credit default swaps and stock markets in the US based on TE from the aspects of intra-structures and inter-structures. Recently, Jang et al. [36] studied the causal relationship between Bitcoin, gold, S&P 500 index, and US dollars using TE, and Yue et al. [37,38] analyzed information transfers between stock market sectors in China and compared between the US and China stock markets. These prior studies can support our idea to use TE as the measure of causal relationships.

Based on the entropy concepts mentioned earlier, conditional entropy can be defined as the expected value of the entropies of the conditional distributions that are averaged over the conditioning random variable. Especially, the conditional entropy of a discrete random variable Y given a discrete random variable X can be expressed as:

(4) $$H\left(X|Y\right)=-\underset{x\;\in X,\;y\;\in Y\;}{\sum }p\left(x,\;y\right)\log_{2}\frac{p\left(x,\;y\right)}{p\left(y\right)}.$$

**Figure 3:**
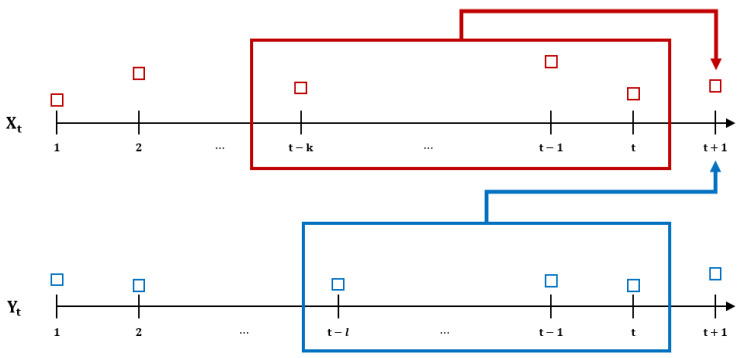
Schematic representation of transfer entropy.

Based on the above definition, we can define the general form of (k, l)-history TE between two discrete time series $X_{t}$ and $Y_{t}$ for $x_{t}^{\left(k\right)}=\left(x_{t},\;..,{\;x}_{t-k+1}\right)$ and $y_{t}^{\left(l\right)}=\left(y_{t},\;..,{\;y}_{t-l+1}\right)$. The general (k, l)-history transfer entropy can be expressed as follows [28]:

(5) $$\begin{array}{ll} {\;\mathrm{TE}}_{Y\to X}^{\left(k,l\right)}\left(t\right) & =H\left(X_{t+1}{|X}_{t},\;\ldots ,{\;X}_{t-k+1}\right)-H\left(X_{t+1}|X_{t},\;\ldots ,{\;X}_{t-k+1},{\;Y}_{t},\;\ldots ,{\;Y}_{t-l+1}\right)\\ & =\underset{i}{\sum }p\left(x_{t+1},{\;x}_{t}^{\left(k\right)},{\;y}_{t}^{\left(l\right)}\right)\log_{2}{p(x}_{t+1}{|x}_{t}^{\left(k\right)},-\underset{i}{\sum }p\left(x_{t+1},{\;x}_{t}^{\left(k\right)},{\;y}_{t}^{\left(l\right)}\right)\log_{2}{p(x}_{t+1}{|x}_{t}^{\left(k\right)})\\ & =\underset{i}{\sum }p\left(x_{t+1},{\;x}_{t}^{\left(k\right)},{\;y}_{t}^{\left(l\right)}\right)\log_{2}\frac{p(x_{t+1}|x_{t}^{\left(k\right)},{\;y}_{t}^{\left(l\right)})}{p(x_{t+1}|x_{t}^{\left(k\right)})}, \end{array}$$

where $i=\left\{x_{t+1},{\;x}_{t}^{\left(k\right)},{\;y}_{t}^{\left(l\right)}\right\}$. ${\mathrm{TE}}_{Y\to X}^{\left(k,l\right)}\left(t\right)$ is nonnegative, and for stationary processes we can drop t, which is the time dependency argument. Accordingly, ${\mathrm{TE}}_{Y\to X}^{\left(k,l\right)}\left(t\right)$ is the information about the future state of $X_{i}$ which can be obtained by subtracting information retrieved from only $X_{t}^{\left(k\right)}$ from information gathered from both $X_{t}^{\left(k\right)}$ and $Y_{t}^{\left(l\right)}$. The schematic representation of transfer entropy is in Figure 3.

In this study, we focused on the TE under the following conditions of two lags k=l=1, which is commonly selected because these settings about lags can be safely assumed on the weak form of the efficient market hypothesis (EMH) and the random walk behavior of stock prices [34,36]. Then, we can express the equation of (1,1)-history TE as follows:

(6) $$\begin{array}{ll} {\mathrm{TE}}_{Y\to X}^{\left(1,1\right)}\left(t\right) & =\underset{i}{\sum }p\left(x_{t+1},{\;x}_{t},{\;y}_{t}\right)\log_{2}\frac{p\left(x_{t+1}{|x}_{t},{\;y}_{t}\right)}{p\left(x_{t+1}{|x}_{t}\right)}\\ & =\underset{i}{\sum }p\left(x_{t+1},{\;x}_{t},{\;y}_{t}\right)\log_{2}\frac{p\left(x_{t+1},{\;x}_{t},{\;y}_{t}\right)p\left(x_{t}\right)}{p\left(x_{t+1},{\;x}_{t}\right)p\left(x_{t},{\;y}_{t}\right)}, \end{array}$$

where $i=\left\{x_{t+1},{\;x}_{t},{\;y}_{t}\right\}$.

However, TE has a disadvantage of finite size effects caused by samples. To complement this demerit, we used ETE in this study. To calculate the effective transfer entropy between two time-series data, we shuffled each element of time-series randomly and designated it as the new time-series sets. Then, we obtained the transfer entropy from this selected time-series called *randomized transfer entropy (RTE)* and subtracted RTE from the originally calculated TE to eliminate noises. This procedure destroys the time-series dependencies of Y and the statistical dependencies between Y and X. Then, the result of this subtraction is ETE, which is as follows. To assess the statistical significance of estimated TE values, we adopt a Markov block bootstrap method proposed in Dimpfl and Peter [31] and tried an average of 100 shuffles from each calculation of ETE. With time delay k and l, the ETE from two random variables $X_{t}^{\left(k\right)}=\left(X_{t},\;..,{\;X}_{t-k+1}\right)$ and $Y_{t}^{\left(l\right)}=\left(Y_{t},\;..,{\;Y}_{t-l+1}\right)$ can be defined as:

(7) $$E{\mathrm{TE}}_{Y\to X}^{\left(1,1\right)}\left(t\right)={\mathrm{TE}}_{Y\to X}^{\left(1,1\right)}\left(t\right)-{\mathrm{RTE}}_{Y\to X}^{\left(1,1\right)}\left(t\right)={\mathrm{TE}}_{Y\to X}^{\left(1,1\right)}\left(t\right)-\frac{1}{M}\overset{M}{\underset{i=1}{\sum }}{\mathrm{TE}}_{Y\to X}^{\left(1,1\right)}\left(t\right),$$

where $Y_{\left(i\right)}$ refers to the shuffled series of Y for all t. We used ETE twice in this study. First, we used ETE to verify causal relationships from ROC of PSI to abnormal returns for selecting politically-themed stocks of politicians. Second, we demonstrated ETE to derive a causal relationship between selected politically-themed stocks to construct each politician’s politically-themed stock network.

To calculate transfer entropy, the data needed to be discretized; thus, we used histogram-based estimation since histogram-based estimation is one of the most common discretization methods for continuous random variables to calculate transfer entropy [29,30,31,32,33,34,35]. We used the histogram-based estimation to calculate entropy measures. In particular, we considered histograms with equally spaced bins. For a continuous random variable, the bin width is determined by selecting an appropriate number of bins in the sample range using mean squared error. Due to their robustness of non-Gaussian data, we decided to use Doane’s equation [39] and the Freedman–Diaconis rule [40]. These two models can complement each other to set a histogram’s bin width and the number of bins in a histogram.

#### 2.3.3. Complex Network Analysis

Complex network analysis is usually used to describe a high degree of interdependence between objects. From this point of view, there can be various network analysis applications to financial systems. Most of the existing studies using network theories concentrate on analyzing correlation, financial stability, and contagion phenomena. Moreover, most of the financial network studies researched network effects rather than network formation [41]. Recently, several papers were published in new research areas such as market analysis, social networks, investment decisions, investment banking, and microfinance. We analyzed politically-themed stock networks on the network level and node level to identify causal influence in politically-themed stock networks.

We used network density (ND) and average ETE to analyze and summarize network dynamics through many periods. These network-level measures have been generally used to analyze stock markets [42,43,44,45] and can effectively illustrate the states of politically-themed stock networks.

(1). Network Density (ND)

The network density (ND) can be defined as the number of edges K to the number of possible connections in a network with N edges. The idea of network density comes from the binomial coefficient. In sum, the ND of the directed graph can be expressed as:

(8) $$\mathrm{ND}=\frac{K}{N\left(N-1\right)}.$$

(2). Average Effective Transfer Entropy

To compare the ETE between politically-themed stock networks, we introduced a measure that describes the average ETE. Let $w_{\mathrm{ij}}$ be elements of N×N weight matrix W (1≤i≤N, 1≤j≤N), where $w_{\mathrm{ii}}=0$ for all 1≤i≤N. Then, the average value is defined as:

(9) $$\overset{-}{W}=\frac{\sum_{i=1}^{N}\sum_{j=1}^{N}w_{\mathrm{ij}}}{N\left(N-1\right)}.$$

We compared the strength of causal relationships at the network level with this measure.

There are many kinds of node-level measures to analyze networks. Especially, the concept of centrality is important in node-level network analysis. There are many types of centrality measures, and we focused on centrality measures that indicate direct influences of politically-themed stocks. For this reason, we considered centrality measurements considering the intuitive influence of individual nodes. In other words, degree centrality (DC), node strength (NS), and PageRank (PR) are the methods used to analyze politically-themed stock networks based on ETE [46]. To compare with other politically-themed stock networks’ results, we used the normalized versions of node-level measures.

(3). Degree Centrality

In unweighted directed networks, degree centrality represents the total number of edges through which a node is connected with other nodes. However, nodes of weighted directed networks have either ingoing edges, outgoing edges, or both. Generally, a node with many other nodes from it is called a *hub*, and a node with many other nodes pointing at it is called an *authority*. Therefore, the degree centrality is analyzed in two measures in weighted directed networks: in-degree (${\mathrm{DC}}_{\mathrm{in}}$) and out-degree (${\mathrm{DC}}_{\mathrm{out}}$). In this study, we divided two original degree centrality measures by N−1 to compare with other politically-themed stock networks on the same scale as we mentioned. In sum, ${\mathrm{DC}}_{\mathrm{in}}$ and ${\mathrm{DC}}_{\mathrm{out}}\;$of the j-th stock can be defined as (9) and (10), respectively:

(10) $${\mathrm{DC}}_{\mathrm{in}}^{j}=\frac{1}{N-1}\overset{N}{\underset{i=1}{\sum }}a_{\mathrm{ij}}$$

(11) $${\mathrm{DC}}_{\mathrm{out}}^{j}=\frac{1}{N-1}\overset{N}{\underset{i=1}{\sum }}a_{\mathrm{ji}}$$

where $a_{\mathrm{ij}}$ are the elements of the adjacency matrix $A\in {\mathbb{R}}^{N\times N}$. For the adjacency matrix A, if there is a link from the stock i to the stock j, $a_{\mathrm{ij}}=1$ and $a_{\mathrm{ij}}=0$ otherwise. Additionally, $a_{\mathrm{ii}}=0\;$for all (1≤i≤N, 1≤j≤N).

(4). Node Strength

In general, node strength is the sum of the weights of links connected to the node. In weighted directed networks, the in-strength is the sum of inward link weights, and the out-strength is the sum of outward link weights. NS represents the influence features in politically-themed stock networks and can be calculated in two measures: in-strength (${\mathrm{NS}}_{\mathrm{in}}$) and out-strength (${\mathrm{NS}}_{\mathrm{out}}$) in the same as degree centrality. We also divided it by N−1 to compare it with other politically-themed stock networks. ${\mathrm{NS}}_{\mathrm{in}}$, and ${\mathrm{NS}}_{\mathrm{out}}\;$of the j-th stock can be defined as (12) and (13), respectively:

(12) $${\mathrm{NS}}_{\mathrm{in}}^{j}=\frac{1}{N-1}\overset{N}{\underset{i=1}{\sum }}w_{\mathrm{ij}}$$

(13) $${\mathrm{NS}}_{\mathrm{out}}^{j}=\frac{1}{N-1}\overset{N}{\underset{i=1}{\sum }}w_{\mathrm{ji}}$$

where $w_{\mathrm{ij}}$ are the elements of weight matrix $W\in {\mathbb{R}}^{N\times N}\;\left(1\leq i\leq N,\;1\leq j\leq N\right)$, where $w_{\mathrm{ii}}=0$ for all 1≤i≤N.

(5). PageRank

PageRank [47] is an algorithm used by Google Search to rank web pages in their search engine results and is one of the famous eigenvector-based metrics that consider all network paths to determine node importance. PageRank has been introduced to rank web pages from the web graph initially. PageRank improved the disadvantages of similar metrics such as the eigenvector centrality and the Katz centrality. PageRank defines a link analysis method for a directed network to evaluate a user’s influence. In finance, PageRank is recently used as the systematic measure [48,49], and stock market analysis [50,51]. Suppose there are N stocks and $A\in {\mathbb{R}}^{N\times N}\;$denotes the adjacency matrix for the politically-themed stock network. In mathematical terms, we can obtain PageRank values of politically-themed stocks as the form of the column vector $r\in {\mathbb{R}}^{N}$ [52]:

(14) $$\left(I-{\alpha A}^{T}D^{-1}\right)r=\left(1-\alpha \right)1.$$

In Equation (14), **I**$\;\in {\mathbb{R}}^{N\times N}$ is the identity matrix, $1\in {\mathbb{R}}^{N}\;$is the column vector whose elements are all one, and **D**$\;\in {\mathbb{R}}^{N\times N}$ is diag($\deg_{i})$ with $\deg_{i}=\max(K_{\mathrm{out}}^{i}$, 1) where $K_{\mathrm{out}}^{i}$ is the number of outward edges started from node i. α is the damping factor, which ranges between zero and one. We set α=0.85, which is the original study’s conventional value [47]. We use the power method to approximate eigenvalues and calculate PageRank values of politically-themed stock networks.

## 3. Empirical Analysis

In this section, we will select and analyze politically-themed stocks empirically. First, we will calculate and select politically-themed stocks of politicians in Section 3.1 and Section 3.2. Then, in Section 3.3, we will construct politically-themed stock networks for verifying leading stocks, which is the first known characteristic of politically-themed stocks in Korea. In Section 3.4, we will analyze the politically-themed stocks’ price changes from the perspective of information flows and network dynamics. After checking the price change of politically-themed stocks, we will develop an investment strategy using politically-themed stocks and back-test the developed strategy. These final two sections are for finding out the second characteristic of politically-themed stocks in Korea: the strong price fluctuation with positive CAR effect.

### 3.1. Calculating the Politician Sentiment Index (PSI)

As we mentioned in 2.2.2, we randomly collected another 35,019 articles from the political section from 2015 to 2020, labeled −1 and 1 to them based on sentiment lexicons, and used them as training data. Additionally, a total of 216,843 text data related to 12 politicians were used as test data to calculate the PSI. Keyword search volume data are based on daily search volume data from Naver Trends and Google Trends.

**Figure 4:**
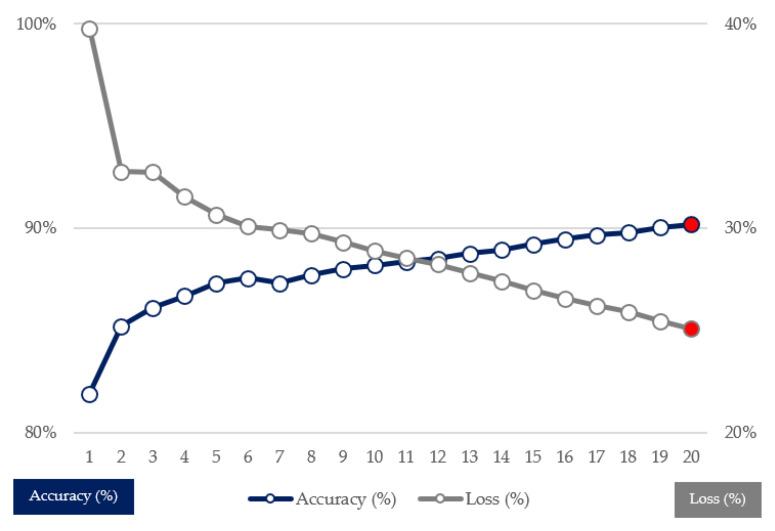
Accuracy curve and loss curve of the trained model.

We used a bi-directional LSTM model for conducting sentiment analysis. An LSTM layer with 128 internal units was used to train the machine-learning model. The activation function was a sigmoid function because it is the appropriate activation function for the binary classification problem, such as sentiment analysis. The maximum epoch, the optimizer, and the loss function were 50, Adam [53] with the 0.001 learning rate and binary cross-entropy, respectively. As a result of training the model, the model’s accuracy after 20 epochs was 90.21%, using the early-stopping method for preventing overfitting. The final epoch was 20 by the result of using the early-stopping method. The accuracy and loss curves of the trained model can be found in Figure 4.

**Figure 5:**
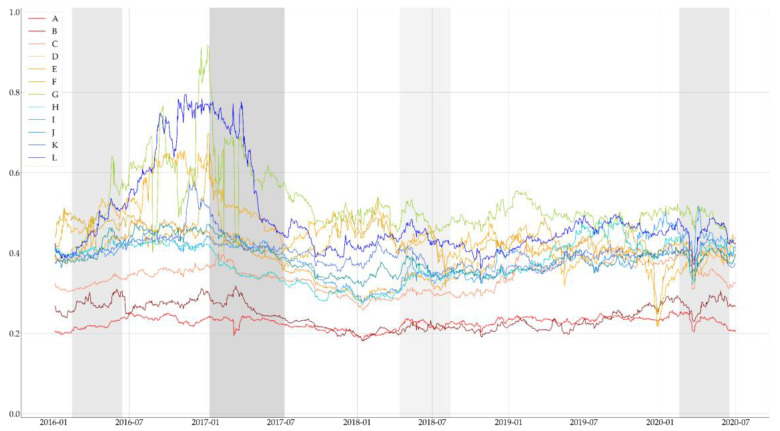
Politicians’ daily PSIs in the research period.

After combining the results of sentiment analysis and politicians’ search volume data using Equation (3), we obtained the politicians’ PSI. If all articles collected on a particular date are classified positively, they have a value of 1 at the date. Additionally, if all articles collected on a particular date are classified negatively, they have a value of 0 at the date. We illustrated the PSI in Figure 5 and marked out grey-colored bars, which represent before and after a month of main political events, which we aforementioned in Table 1. The saturation of the gray-colored bar means the political significance of political events. As we mentioned, the PSI of politicians belonging to left-wing parties is high overall, starting with the change of the impeachment of the Eighteenth President, which can be interpreted as the result of the Korean political regime’s transfer to politicians belonging to left-wing parties. Taking the political power of politicians belonging to left-wing parties after the nineteenth presidential election, the overall strength continues, but it tends to fall slightly lower than the time around the impeachment of the Eighteenth President.

The dataset, which includes 12 politicians’ ROC of the PSI, comprises 1101 rows for each politician. Table 3 shows the descriptive statistics in accordance with the ROC of PSI. We checked elementary statistics and performed the Shapiro–Wilk test and Jarque–Bera test for normality, the Ljung–Box test with 10-order serial autocorrelation in squared returns, and the White test for homoskedasticity, the same as the abnormal return data.

Our statistical test results for ROC of PSI show departure from normality based on the Shapiro–Wilk and Jarque–Bera test at all three significance levels. Additionally, all 12 ROC of PSI data were also stationary according to the augmented Dickey–Fuller (ADF) test, Phillips and Perron (PP) test, and Kwiatkowski–Phillips–Schmidt–Shin (KPSS) test. We included the ADF test results as the representative of those three stationary tests for the same reason in Section 2.2.4.

Moreover, we fitted the 12 ROC of PSI data to determine the best-fitting distribution among well-known continuous distributions. This experiment was conducted using Python ‘*SciPy*’ package’s all available distributions and the criterion for selecting the best-fitting distribution is Kullback–Leibler divergence loss. We can find that all of the 12 ROC of PSI data are not normally distributed, and the results are in Table 4. These results give justification for our purpose to use TE once again.

### 3.2. Selecting Politically-Themed Stocks

We measured the information flow between the PSI and politically-themed stock candidates, including hidden cause–effects based on the ETE with k=l=1 case for assuming important hypotheses of stock markets aforementioned in Section 2.3.2.

**Figure 6:**
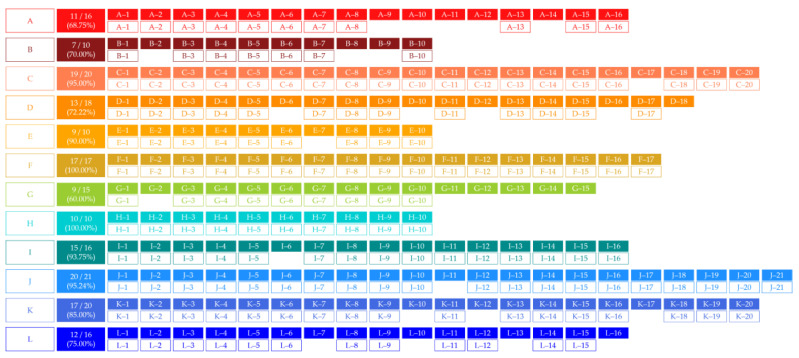
Politically-themed stock candidates and selected politically-themed stocks.

**Figure 7:**
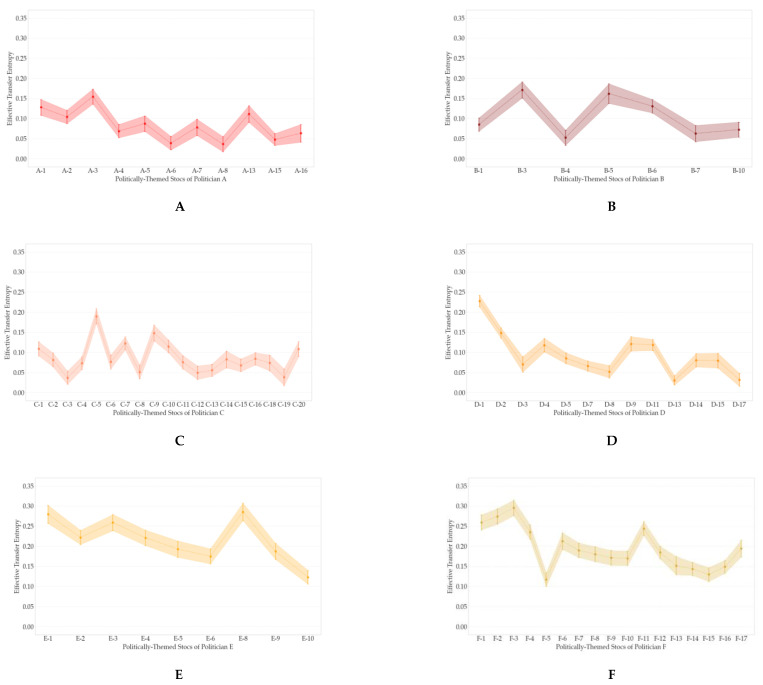

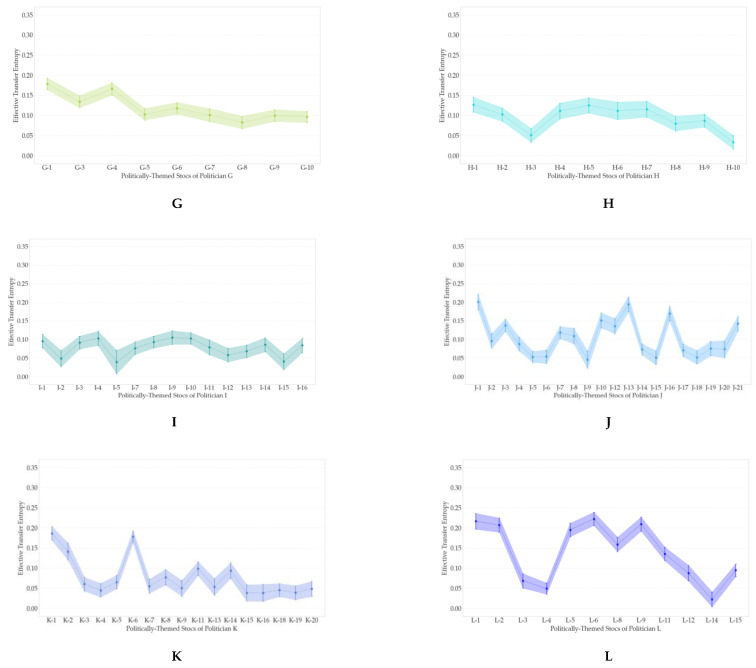
The ETE from ROC of the PSI to abnormal returns of politically-themed stocks. Note: the length of error bars means a 95% confidence interval for the mean values of ETE. The characters of subfigures (**A**–**L**) mean the politicians’ assigned alphabet for de-identification.

In Table 5, there are the number and proportion of stocks that have ETE-based causal relationships from the ROC of PSI to abnormal returns. We inferred that ETE detected additional causal relationships between the ROC of PSI and abnormal returns from the number of stock candidates with statistically significant causal relationships in Table 5. From the results in Section 3.1, we chose politically-themed stocks that are statistically significant at α=0.01, considering the robustness of the results. Additionally, we excluded stocks whose TE values were zero when they were rounded to the fourth decimal place. As a result, the 159 stocks, which are 84.13% of politically-themed stock candidates, were selected as the politically-themed stocks for constructing politically-themed stock networks. The selection results containing 12 politicians and their ETE values are shown in Figure 6 and Figure 7, respectively. In sum, unlike known facts and qualitative selection methodologies of prior research, not all politically-themed stock candidates are selected as politically-themed stocks based on this study’s quantitative selection methodologies. Additionally, greater than or equal to 60% of politically-themed stock candidates were designated as politically-themed stocks of politicians, as shown in Figure 6. Additionally, the ratio of selected politically-themed stocks of politicians to politically-themed stock candidates of 12 politicians did not proportionate to the number of politically-themed stock candidates of politicians.

Based on the above results, we conducted statistical analyses to measure correlations between the politically-themed stocks’ de-identified numbers and the rank of ETE amounts of politically-themed stocks. As we mentioned, the politically-themed stock’s de-identified number means the rank of politician-related search volumes in the particular politician’s politically-themed stocks. We sorted the politically-themed stocks’ ETE in descending order and ranked them to measure the correlation between the two ranks. We used the Spearman rank coefficient and Kendall rank correlation coefficient to identify the monotonic relationships between politician-related search volumes and ETE amounts. You can see the example of measuring two rank correlations measures between Politician H’s politically-themed stocks’ de-identified numbers and ETE rank values in descending order in Table 6. 

**Figure 8:**
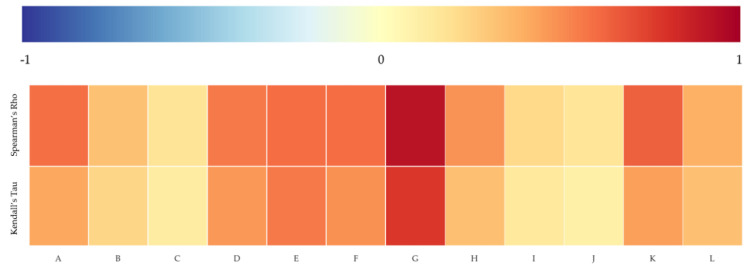
The rank correlation between politician−related search volumes and ETE of politically-themed stocks.

As a result, there are significant positive monotonic relationships between the ranks of politically-themed stocks and ETE, as shown in Figure 8. We may consider these results as evidence to support the idea that a politically-themed stock known to have a strong relationship with a politician can be more affected by the politician’s reputation. In other words, the stocks whose politician-related search volumes are large and known as *leading stocks* of politically-themed stocks are strongly linked to politicians.

### 3.3. Developing Politically-Themed Stock Networks

**Figure 9:**
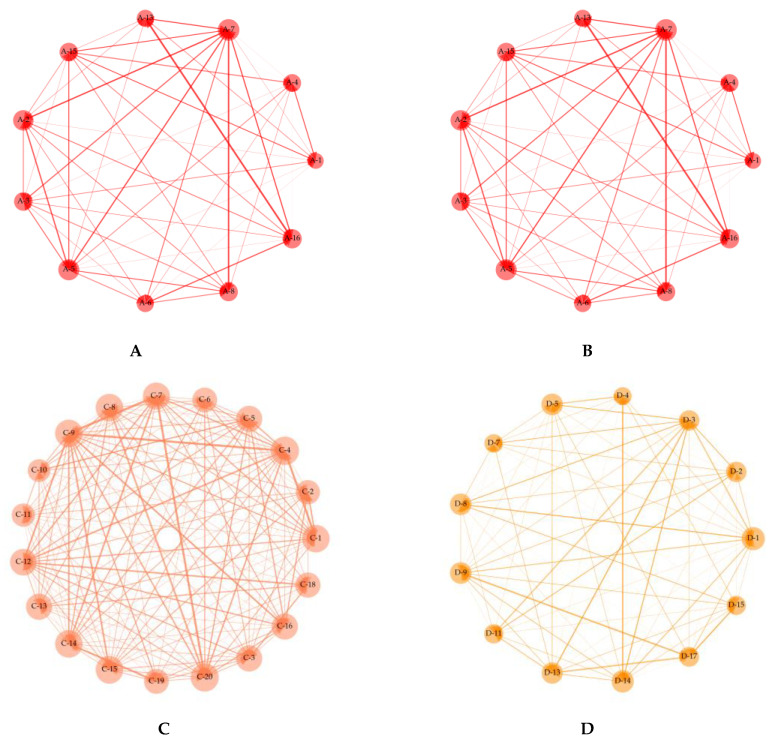

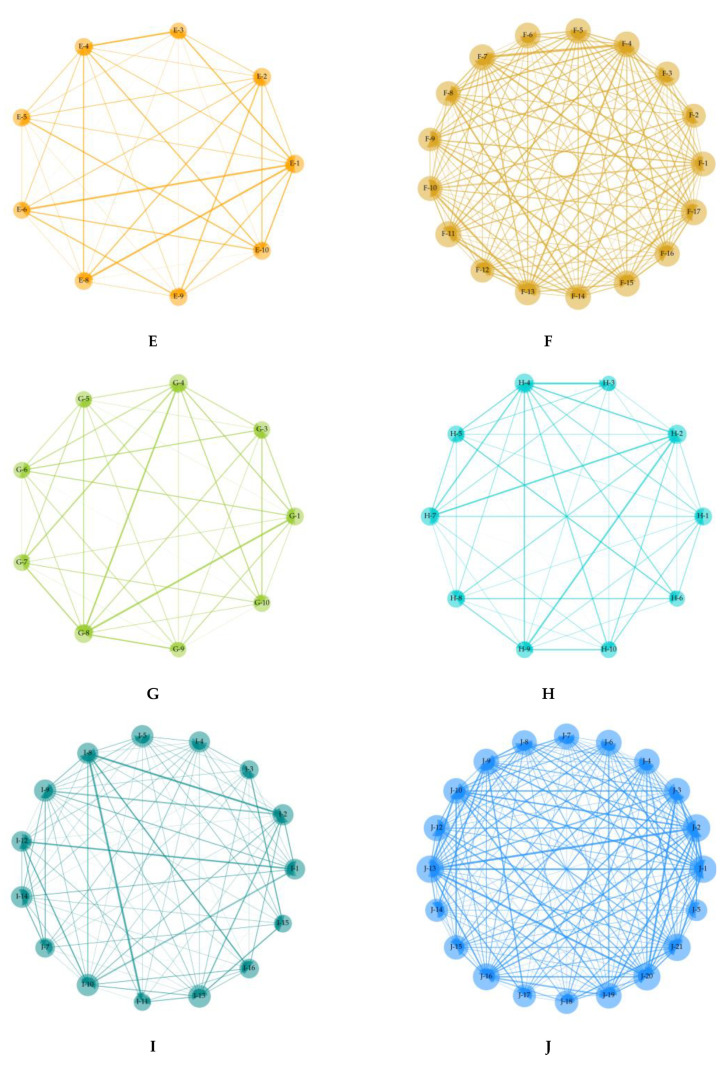

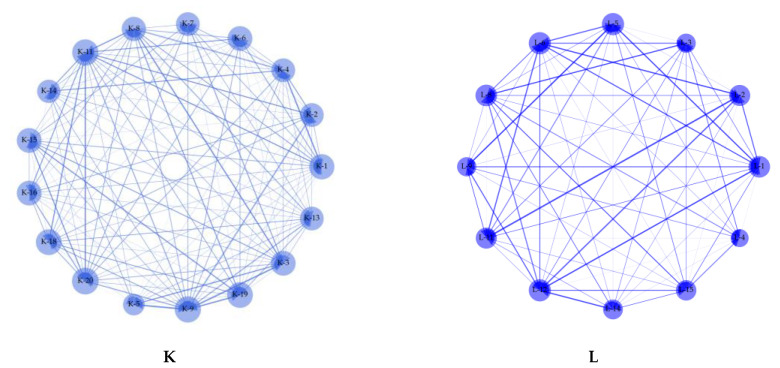
Politically-themed stock networks in the whole research period. Note: The characters of subfigures (**A**–**L**) mean the politicians’ assigned alphabet for de-identification.

We illustrated politically-themed stock networks based on ETE using weighted directed networks. The number of statistically significant stocks generated at each significance level is given in Table 7. As a result, we finally illustrated politically-themed stock networks of 12 politicians with statistically significant ETE at significance level α=0.01, which is demonstrated in Figure 9. We chose the significance level by referring to previous research results, including TE and ETE [23,24,25,26,27,28,29,30,31,32]. In Figure 9, the direction of arrows represents the causal link and the width of arrows represents the size of ETE. Additionally, the size of nodes was proportionate to the summation of two degree-centrality measures, and the width of edges was in proportion to the summation of the estimated ETE.

**Figure 10:**
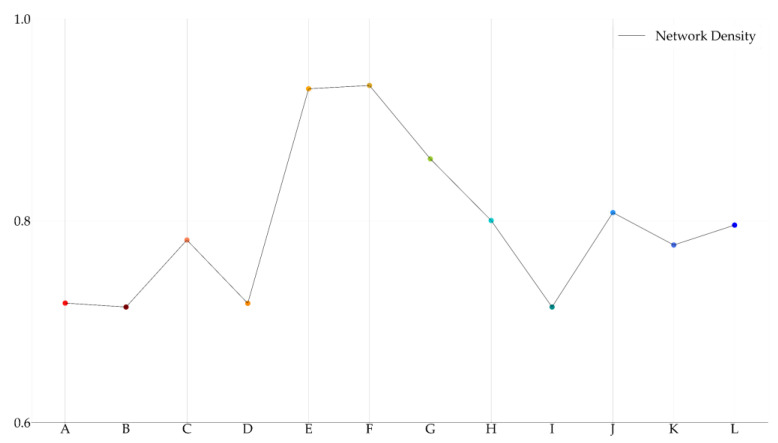
Network density of politically-themed stock networks based on ETE.

**Figure 11:**
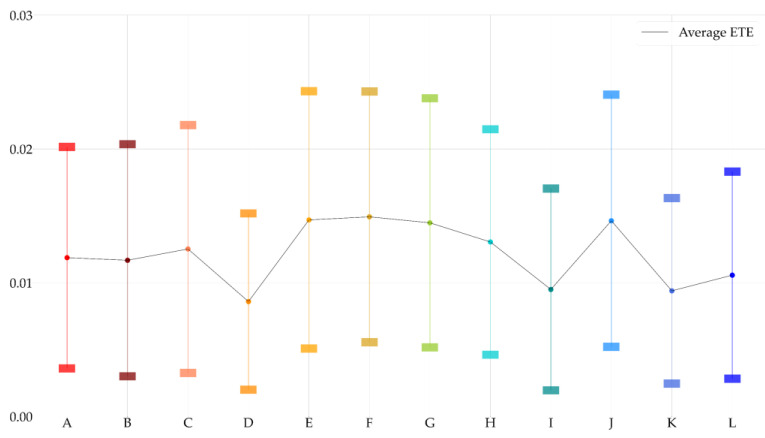
Average ETE values of politically-themed stock networks. Note: the length of error bars means a 95% confidence interval for the mean values of ETE.

Based on Figure 9, we demonstrated network-level analysis, as mentioned in Section 2.3.3. No politically-themed stock is isolated in all politically-themed stock networks. This result means that all selected politically-themed stocks are linked to other politically-themed stocks and supports the notion that selected politically-themed stocks can be considered as the particular politician’s politically-themed stocks statistically. The average network density of all politically-themed stock networks was 0.7981. In other words, 79.81% of all possible edges exist. This indicates that politically-themed stock networks are dense networks, and various causal relationships are included in politically-themed stock networks. The network density values and average ETE values of politically-themed stock networks are demonstrated in Figure 10 and Figure 11.

**Figure A1:**
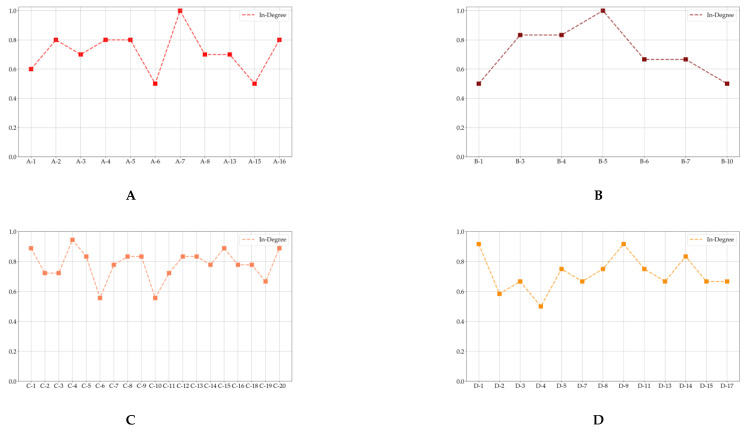

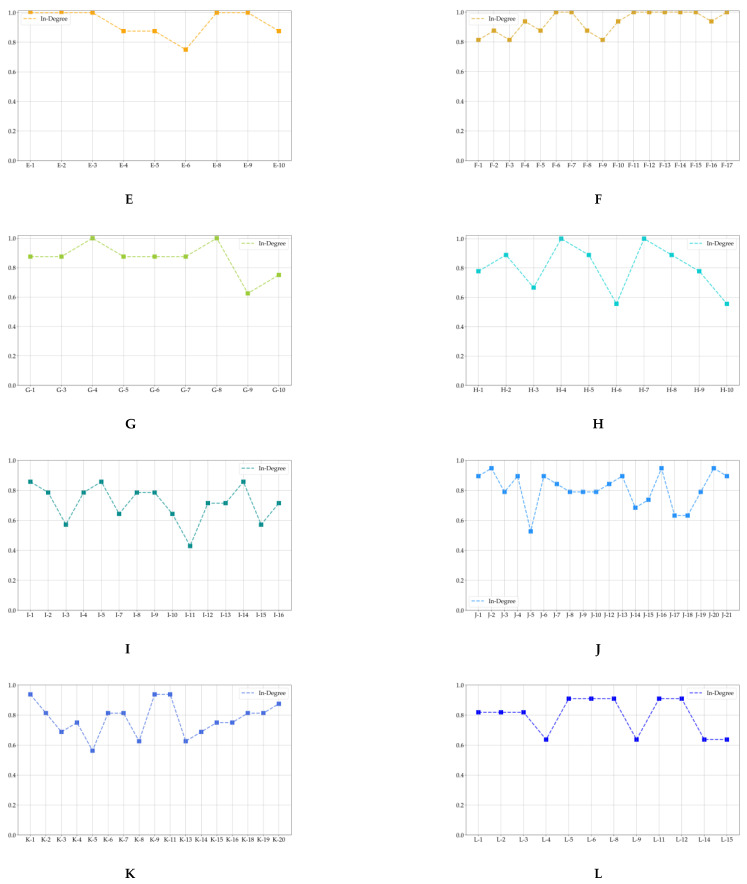
In-degree of politically-themed stocks. Note: The characters of subfigures (**A**–**L**) mean the politicians’ assigned alphabet for de-identification.

**Figure A2:**
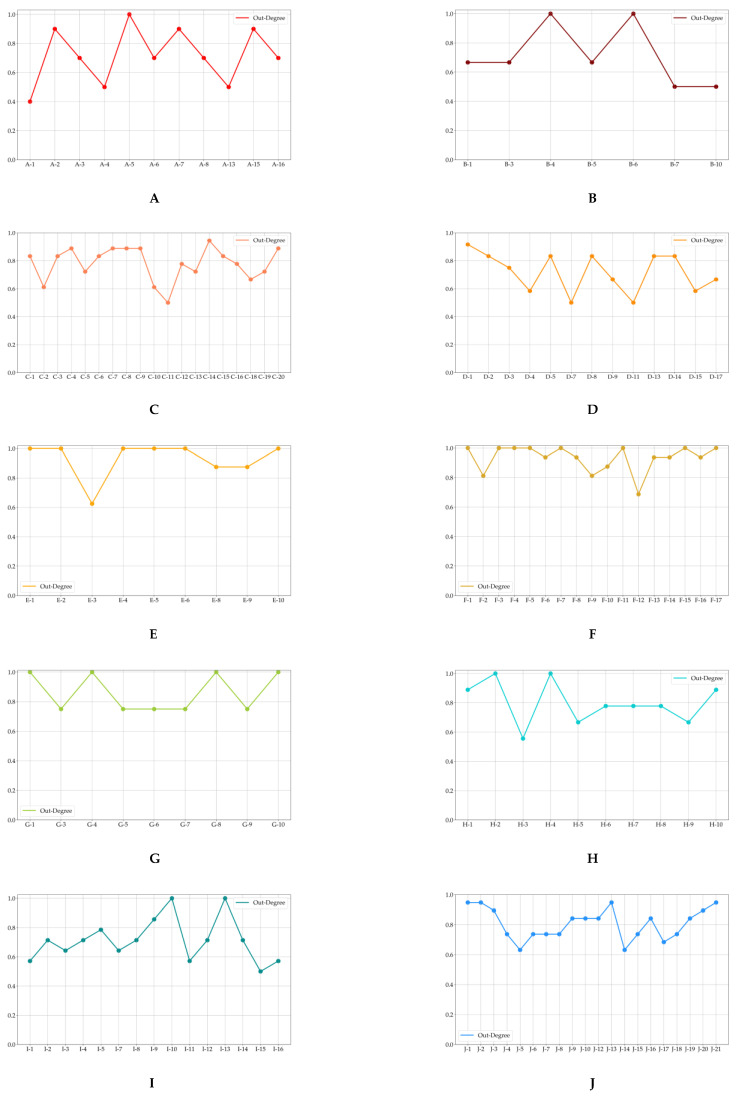

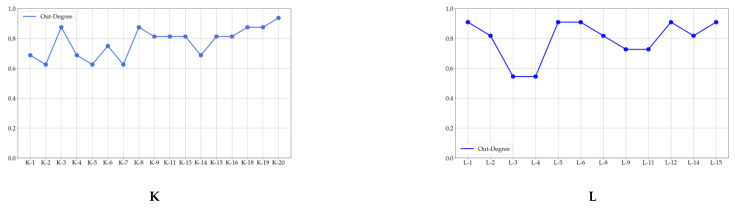
In-degree of politically-themed stocks. Note: The characters of subfigures (**A**–**L**) mean the politicians’ assigned alphabet for de-identification.

**Figure A3:**
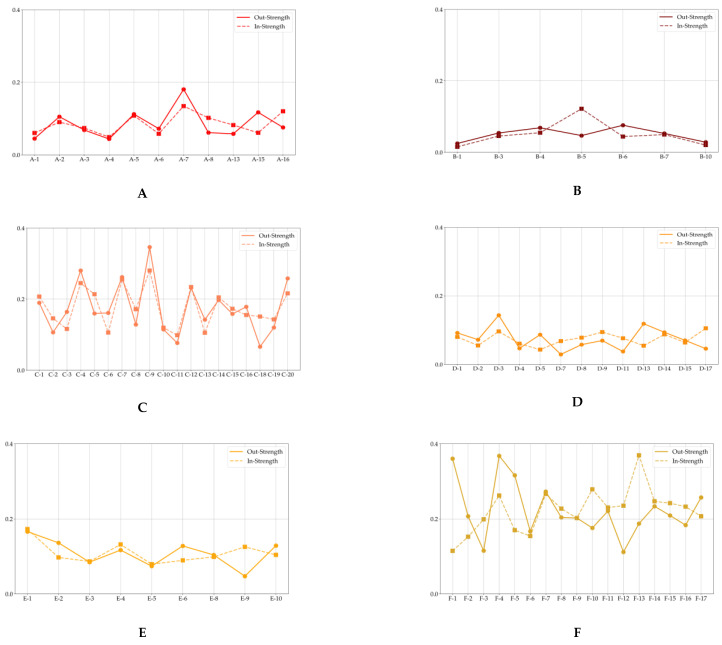

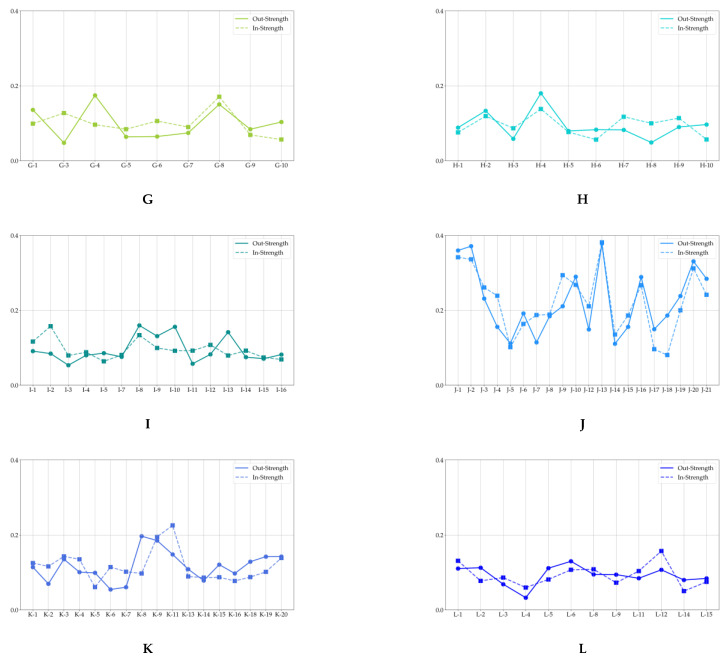
In-strength and out-strength of politically-themed stocks. Note: The characters of subfigures (**A**–**L**) mean the politicians’ assigned alphabet for de-identification.

**Figure A4:**
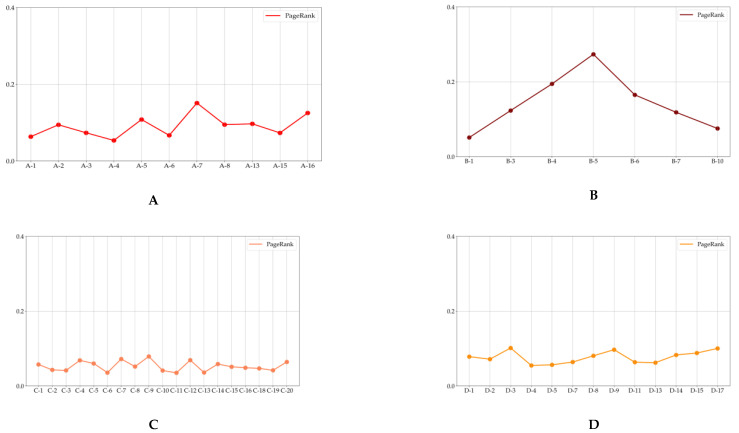

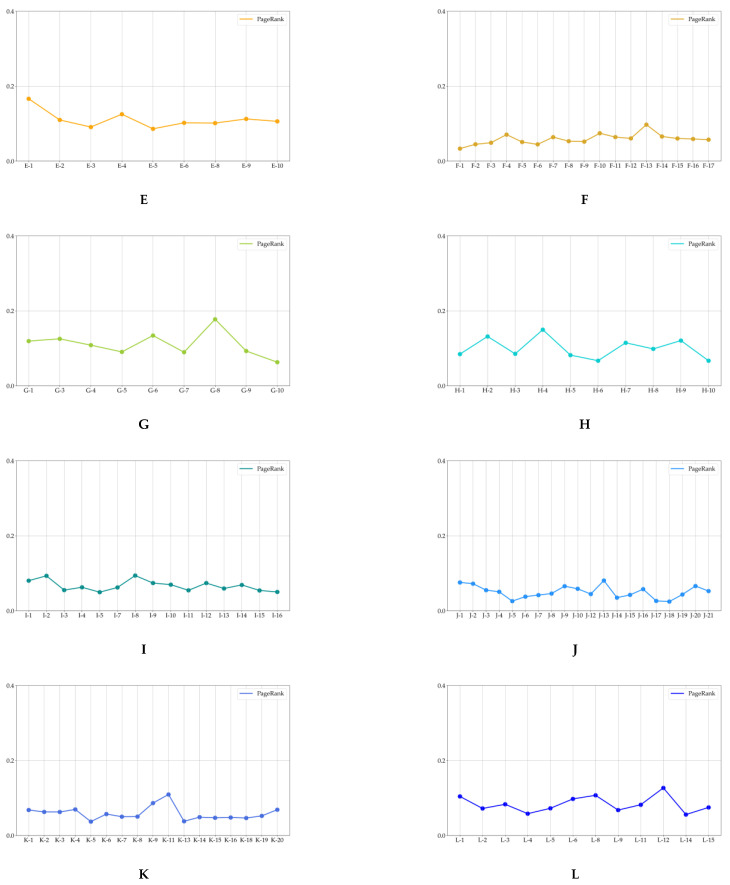
PageRank of politically-themed stocks. Note: The characters of subfigures (**A**–**L**) mean the politicians’ assigned alphabet for de-identification.

From the fact that more than three-quarters of causal relationships are statistically significant, we tried to analyze the impacts of individual stocks in politically-themed stocks in detail. We conducted the node-level network analyses, and their results are shown in Figure A1, Figure A2, Figure A3 and Figure A4 of Appendix B. We divided in-strength plots and out-strength plots due to problems in plotting two attributes that overlap.

From Figure A1, Figure A2, Figure A3 and Figure A4, we can identify the existence of leading stocks in politically-themed stock networks from unequal values of in-degree, out-degree, in-strength, out-strength, and PageRank. Additionally, all in-degree and out-degree centrality values are over 0.5, which means that over 50% of possible connection exists. This result means these politically-themed stock networks can be considered as highly connected networks. These unequal network measure values imply that some price movements of politically-themed stocks influence other stocks’ price movements. Especially, the stocks that have high out-degree, out-strength, and PageRank values can be regarded as leading stocks, which is one of the main characteristics of politically-themed stocks in Korea [6,7,8,9]. These leading stocks act as the leading role of politically-themed stocks in politically-themed stock networks. Table 8 includes the information of the politically-themed stocks which can be the leading stocks of politically-themed stock networks based on their out-degree, out-strength, and PageRank values. 

Moreover, if we define a stock whose abnormal returns are heavily influenced by other politically-themed stocks as the *following stock*, then the following stocks of politically-themed stock networks are also included. The selection criteria of leading stocks and following stocks are that they meet the top five for all mentioned network measures in each politically-themed stock network. As a result, these selected leading stocks and following stocks have many intersections. In detail, 47.27% of selected leading stocks or following stocks based on network measures act as both leading and following stocks. These selected stocks can be interpreted as having very close relationships with other politically-themed stocks in their politically-themed stock networks. Additionally, if politically-themed stocks in a particular politically-themed stock network have very similar node strength and centrality measure values, no stock is classified as leading stocks or following stocks based on our criteria. In other words, if all the stocks at the top-ranked stocks of each network measure values are all different, there can be no leading stocks or following stocks in a politically-themed stock network. However, two or more stocks in all politically-themed networks are classified as leading stocks or following stocks. Therefore, this result indicates that these stocks play a very important role in the politically-themed stock network from the perspective of node-level network measures. Furthermore, these selected stocks could be considered as the first-ordered investigating stocks to check the violation of stock market regulations from the perspective of financial regulatory authorities.

### 3.4. Network Dynamics Before and after Political Events

In this section, we checked the network dynamics before and after political events to find evidence of politically-themed stocks’ dramatic price changes around political events. We set up six experimental points. Five experimental points are the timepoints that five political events occurred in the research. The other experimental point is the control point in time to compare with the other five political event periods’ results. We considered one control point in time and selected it on April 17, 2019, because the third Wednesday in April is Korea’s official election period if there is an election to be held in that year. In sum, the research periods for conducting network dynamics analysis before and after political events are shown in Table 9.

**Figure 12:**
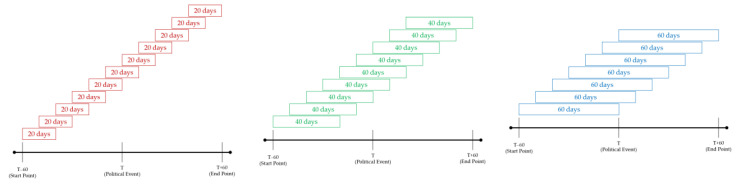
Applying sliding window methods in this study.

Various studies show that meaningful changes occurred before and after political events in Korea, like positive CAR before political events and losses after political events [6,7,8,9,54]. To identify politically-themed stock networks’ change of dynamics, we used sliding window methods to detect politically-themed stock networks’ network dynamics with the 10-day step size before and after political events. We set the range for observing their network dynamics as 60 trading days before and after political events with reference to prior studies [1,2,3,4,5,6,7,8,9]. Then, we developed three window sizes: a 20-day window, a 40-day window, and a 60-day window to examine the effects of politically-themed stocks from various angles by setting up different window sizes, shown in Figure 12 [9].

**Figure 13:**
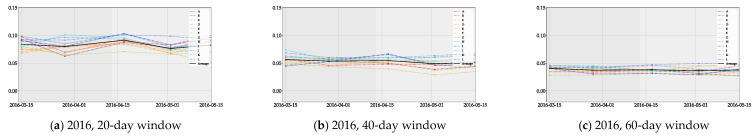

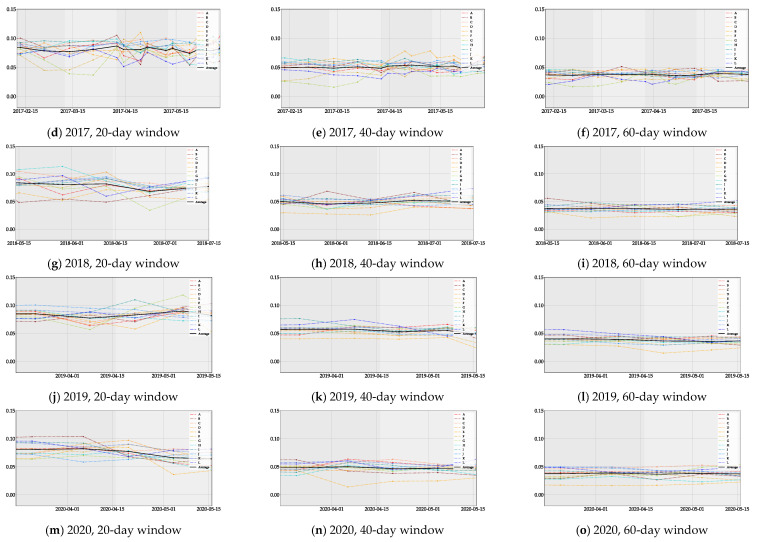
The evolution of average ETE values before and after political events.

As a result, we can obtain the insight that the average ETE values with 20-day windows are clearly larger than those of 40-day windows and 60-day windows based on Figure 13. We can obtain the insight that the average ETE values with 20-day windows show more volatile movements with stronger asymmetry information flows than others. Moreover, most average ETE values are gently decreasing before political events and increasing after political events. The decreasing trends before political events can be interpreted as the change from information flows due to leading politically-themed stocks to simultaneous movements from the perspective of daily abnormal returns. As a result, these trends of average ETE values can be elucidated that causal relationships go back to their normal states after political events. This result can be translated to the unique movements derived from investors expecting profits before and right after political events. These periods are commonly known as the most profitable periods [6,7,8,9,54].

In conclusion, we can find out the trends for increasing ETE before and after political events. For the control period (Period 5), the results showed the opposite movement of the political events period or oscillated without apparent direction, making it hard to define. Therefore, the increasing ETE around political events may support those rapid stock movements, which is the characteristic of politically-themed stocks in Korea. This phenomenon can be translated that politically-themed stocks affected each other stronger and have more information flows than normal periods.

### 3.5. Investment Strategy Based on PSIs and Politically-Themed Stock Networks

Based on the previous papers about politically-themed stocks and the results of previous sections [1,2,3,4,5,6,7,8,9,54], we obtained the insight of dramatical price changes of politically-themed stocks with a positive CAR effect of politically-themed stocks occurring around political events. Especially, the size of politically-themed stocks’ positive CAR effect occurred more positively in Korea if the politician receives benefits from political events [6,7,8,9]. Based on these results, we developed an investment strategy using the PSI and politically-themed stock networks as the class of assets [55] to verify the possibility of benchmarking KOSPI and KOSDAQ, which are the major market indices of the stock markets in Korea. The strategy can be described as performing the following steps:

***Step*** ***1***: Check the daily ROC of the PSI and pick the largest increasing PSI.
***Step*** ***2***: Select the politically-themed stock networks of the politician whose PSI are selected in the first stage.
***Step*** ***3***: Optimize the portfolio with the stocks in the selected politically-themed stock networks.

To apply this strategy, we assumed the daily rebalancing period and the weights of stocks were obtained from the solution of portfolio optimization problems to maximize the Sharpe ratio. Maximizing the Sharpe ratio in the portfolio optimization problem is one of the most commonly used portfolio optimization problems. The Sharpe ratio has advantages that consider the return and risk in the objective function simultaneously and can be computed directly from any observed time-series of returns regardless of additional information from stocks. The portfolio optimization problem that maximizes the Sharpe ratio is as follows [56,57]:

(15) $$\begin{array}{c} \max\;\frac{w^{T}\mu -r_{f}}{\sqrt{w^{T}\Sigma w}}\\ \mathrm{subject}\;\mathrm{to}.\;1^{T}w=1,\;w\geq 0 \end{array}$$

where $w\in {\mathbb{R}}^{N}$ is the column vector consists of the weight of assets, $\mu \in {\mathbb{R}}^{N}$ is the mean return vector, **Σ**$\in {\mathbb{R}}^{N\times N}$ is the covariance matrix. The column vectors $r_{f}\in {\mathbb{R}}^{N}$ and $1\in {\mathbb{R}}^{N}$ have elements all consisting of the risk-free rate and one, respectively. The first constraint means that the sum of portfolio weight is one, and the second one implies that short-selling is not allowed, which is hard to carry out by individual investors. We used the stock return data from before 20 trading days to calculate the average return vector and the covariance matrix. The South Korea 10 Years Government Bond was used as the risk-free rate [58]. We also considered transaction costs to prevent overestimating performance, critical to the daily rebalancing period portfolio optimization problem. In other words, we made an assumption that we sell all of our stocks, which we held at the before period, and buy new stocks when we rebalance our portfolio. This assumption was applied to the case that the selected politically-themed stock network is the same for several days in a row. Therefore, the cumulative return data of the portfolio are the lower bound of the portfolio’s performance. We also optimized the portfolio from all politically-themed stocks without considering the PSI and to where their networks belong. Additionally, we considered the reformulation version considering computational times and achieving the convexity of the objective function [59].

**Figure 14:**
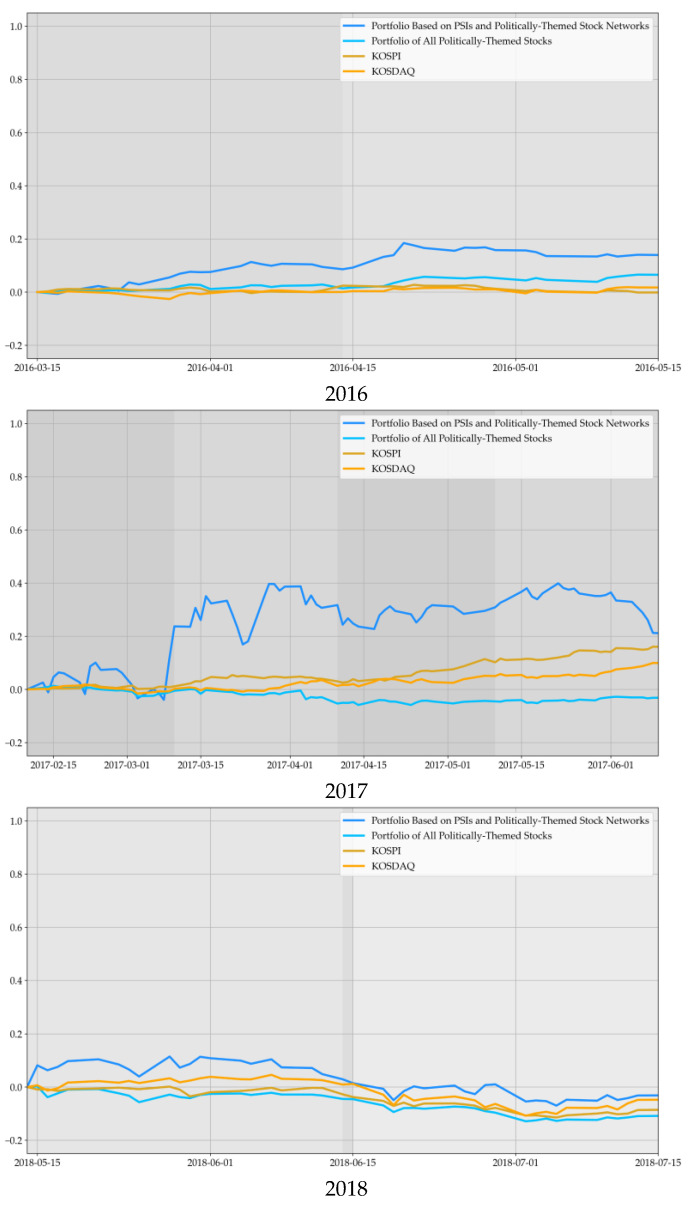

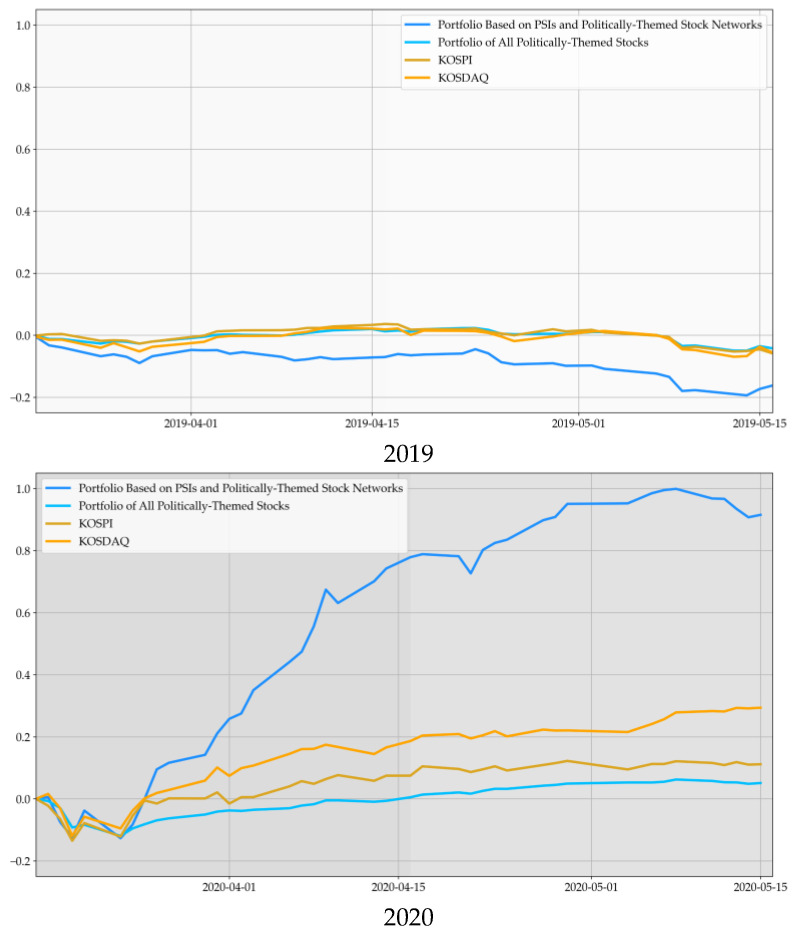
Portfolio performance during the research periods.

The portfolio with our investment strategy can obtain additional profit over both market indices before and around the political events, as shown in Figure 14. Moreover, the portfolio’s performance based on the PSI and politically-themed stock networks increased sharply in the presidential and legislative elections, which are the most important political events in Korea. In 2017, there were two significant political issues: the impeachment of the Eighteenth President and the nineteenth presidential election; we can identify the upward tendency in the pre-political event and the downward trend in the post-political event more accurately. Before and after the control point in time, the portfolio’s performance using the PSI and politically-themed stocks was worse than any other portfolio. Despite downtrends after political events, the portfolio’s final performance using the PSI and politically-themed stocks exceeded the cumulative returns of KOSPI and KOSDAQ through the whole research period.

Moreover, the portfolio of all politically-themed stocks without using the PSI and the derived strategy always showed the lowest performance in almost all research periods. These results implicate that the investment in politically-themed stocks without any strategy is precarious and fragile. This low performance is originated from the fact that most politically-themed stocks have small caps, and their financial stabilities and intrinsic values are unverified.

After all, these results can verify the positive CAR effect before and after political events of prior studies [1,2,3,4,5,6,7,8,9]. Additionally, eccentric movements and changes of politically-themed stocks occurred before and after political events simultaneously. These results may be considered as evidence of the Korean stock markets’ instability, and is similar to previous studies of politically-themed stocks in Korea from the perspective of the stability of the Korean stock market [6,7,8,9,54].

## 4. Discussion

In this study, we developed a novel method for selecting politically-themed stocks by verifying information flows from positive and negative reputations of politicians to their stock returns. After selecting politically-themed stocks, we tried to verify two characteristics of politically-themed stocks using politically-themed stocks: the existence of leading stocks and the occurrence of positive CAR effect with strong price fluctuation around political events.

We set the research period from 1 January 2016, to 30 June 2020. We selected this period because all three primary elections in Korea were held, and a unique political situation (impeachment of the Eighteenth President) is included in this research period. We selected 12 politicians with experience in presidential elections or politicians who had been mentioned as strong candidates in presidential elections for the research periods [1,2,3,4,5,6,7,8,9]. All 12 selected politicians were de-identified using letters A to L.

Using collected politician-related search volume data, we designated the stocks whose search volumes were more than 1% of the search volume of the most searched stocks as the politically-themed stock candidates from the search results. In sum, we obtained 189 politically-themed stock candidates. A range of 10 to 21 stocks were allocated as politically-themed stock candidates for 12 politicians. Like politicians, 189 politically-themed stock candidates were de-identified. 

After collecting data, we first calculated abnormal returns based on the market model. Each abnormal return dataset consisted of 1101 daily abnormal return data of 189 politically-themed stock candidates. We checked statistical tests for abnormal return datasets. We summarized statistical test results in Figure 2, and they show that our abnormal return dataset exhibits departure from normality and non-stationarity.

We conducted sentiment analysis to calculate the PSI in this study. We randomly collected another 35,019 articles from the political section from 2015 to 2020, labeled them with sentiment lexicons, and used them as training data. Additionally, we designated 216,843 documents related to 12 politicians to the text data and performed sentiment analysis. An LSTM layer with 128 internal units was used to train the machine-learning model for sentiment analysis. The final accuracy of the machine learning model was 90.21%, as shown in Figure 4. After combining the results of sentiment analysis and politicians’ search volume data, we finally obtained the politicians’ PSI. The calculated PSI is shown in Figure 5. After calculating the ROC of the PSI, we conducted the same statistical tests on the abnormal return data, and found that all the ROC of the PSI data are non-Gaussian and stationary, as shown in Table 3 and Table 4.

To measure the causal relationships between the two non-Gaussian data, we used TE calculated from histogram estimation. TE does not assume normality and linear autoregression between variables, which are the core assumptions of conventional economics [16,17]. This attribute makes it possible to test causal relationships between non-linearly interacting variables. Therefore, it has been widely used in many places to set apart the system’s source variables and target variables [22,54,55,56,57]. In particular, financial markets are complex systems that express collective phenomena based on the interacting individual agents, so TE can better detect inter-causal relationships than the Granger causality test [21,60,61,62]. Especially, we used ETE in experiments because ETE is the complementary measure of transfer entropy in terms of finite-size effects caused by samples. We used the histogram-based estimation to calculate entropy measures

After computing ETE from the ROC of the PSI to abnormal returns, we chose politically-themed stocks that are statistically significant at α = 0.01 considering the robustness of results. Additionally, we excluded stocks whose TE values were zero when they were rounded to the fourth decimal place. As a result, the 159 stocks, which are 84.13% of politically-themed stock candidates, were selected as the politically-themed stocks for constructing politically-themed stock networks. The results of politicians’ politically-themed stocks and their ETE are shown in Figure 6 and Figure 7. As a result, contrary to conventional thought, not all politically-themed stock candidates are selected as politically-themed stocks based on this study’s methodologies. Greater than or equal to 60% of politically-themed stock candidates are designated as politically-themed stocks of politicians, as shown in Figure 6. Additionally, the ratio of selected politically-themed stocks to politically-themed stock candidates did not proportionate to politically-themed stock candidates.

We illustrated politically-themed stock networks based on ETE using weighted directed networks, as shown in Figure 9. Then, we calculated the network density, average values, and frequency distributions to analyze and summarize politically-themed stock networks dynamically at the network level to obtain insights from the politically-themed stock networks. These network-level measures are generally used to analyze stock markets [32,41,42]. From the perspective of node-level network analyses, we focused on centrality measures, which measure politically-themed stocks’ direct influence. For this reason, we considered centrality measurements considering intuitive influence to analyze node-level networks. In other words, in-degree, out-degree centrality, in-strength, out-strength, and PageRank are the methods used to analyze politically-themed stock networks at the node level [48,49]. The overall network density was 0.7981. In other words, 79.81% of all possible edges exist. It indicates that politically-themed stock networks based on ETE are a dense graph, and various causal relationships are included in politically-themed stock networks.

From Figure A1, Figure A2, Figure A3 and Figure A4, we can identify the existence of leading stocks in politically-themed stock networks from unequal values of in-degree, out-degree, in-strength, out-strength, and PageRank. Additionally, almost all in-degree and out-degree values were over 0.5, which means that over 50% of the possible connections were connected. This result means politically-themed stock networks in this study can be considered as highly connected networks. Table 8 includes the information of the politically-themed stocks which can be the leading stocks of politically-themed stock networks based on their out-degree, out-strength, and PageRank values. The selection criteria are those that met the top five for all corresponding network measures in each politically-themed stock network. As a result, these selected leading stocks and following stocks have many intersections. In detail, 47.27% of selected leading stocks or following stocks based on network measures act as both leading and following stocks. These selected stocks can be interpreted as having very close relationships with other politically-themed stocks belonging to politically-themed stock networks. Additionally, these selected stocks could be considered as the first-order-of-investigation stocks to check the violation of stock market regulations from the perspective of financial regulatory authorities.

We can find out the trends for increasing ETE before and after political events. Additionally, we checked the network dynamics before and after political events for finding evidence of politically-themed stocks’ dramatic price changes around political events. We checked those network dynamics using average ETE values of politically-themed stocks in the same politically-themed stock network with sliding window methods. After conducting analyses, we can obtain the insight that the average ETE values with 20-day windows show more volatile movements with stronger asymmetry information flows than others. Moreover, average ETE values are gently increasing after political events.

Based on the previous papers about politically-themed stocks and results of previous sections [1,2,3,4,5,6,7,8,9,54], we obtained the insight that the abnormal returns of politically-themed stocks occurred before and after political events. Especially, the size of politically-themed stocks’ CAR occurred more positively in Korea if the politician receives benefits from political events [6,7,8,9]. From this insight, we developed the investment strategy using the PSI and politically-themed stock networks as the class of assets [55]. Using this investment strategy, we tried to benchmark the KOSPI and KOSDAQ, which are the major market indices of the stock market in Korea. As a result, the portfolio with our investment strategy can obtain additional profit than both market indices before and around the political events, as shown in Figure 14. Moreover, the portfolio’s performance based on the PSI and politically-themed stock networks increased sharply in the presidential and legislative elections, which are the most important political events in Korea. Additionally, there are two significant political issues: the impeachment of the Eighteenth President and the nineteenth presidential election in 2017. We can identify the upward tendency in the pre-political event and the downward trend in the post-political event more accurately in 2017. The optimal portfolio based on all politically-themed stocks without using the PSI and politically-themed stock networks almost always underperformed than other portfolios in the entire research period. In the control period, the portfolio’s performance using the PSI and politically-themed stocks was worse than any other portfolio. Plus, the portfolio of all politically-themed stocks without using tge PSI and the derived strategy always showed the lowest performance in almost all research periods.

## 5. Conclusions

*Politically-themed stocks* are generally defined as stocks that are expected to benefit from policies and politicians. By this definition, there can be two types of politically-themed stocks: *policy-related politically-themed stocks* and *politician-related politically-themed stocks*. Especially, politician-related politically-themed stocks are defined as stocks that are privately related to major stakeholders related to politicians. At this time, it means that the major stakeholders of a particular company have a form of relationship that has no relation with the politicians’ policy direction. Instead of their policy direction, those stakeholders are connected to politicians based on blood ties, regional ties, academic ties, etc. In this research, we focused on the politician-related politically-themed stocks, not on the ones benefiting from the politicians’ policies, but on the ones benefiting from politicians’ connections. Most empirical studies of these politically-themed stocks have claimed that two representative characteristics of politically-themed stocks exist: leading stocks and the positive CAR effect. 

This study attempted to select politically-themed stocks as information flows from the positive or negative reputation of politicians to abnormal returns in order to supplement the methodologies of existing research. In the process, politically-themed stocks were selected as the ones with statistically significant causal relationships from the positive or negative reputation of politicians. We then used the selected politically-themed stocks to validate the two characteristics: the presence of leading stocks and the occurrence of positive CAR effects around political events.

To select politically-themed stocks, we calculated the daily PSI, which means politicians’ daily reputation using politicians’ search volume data and politicians’ sentiment analysis results. Using the calculated PSI, we determined politically-themed stocks based on causal relationships from ROC of the PSI to the abnormal returns derived from the market model between the politically-themed stock candidates using ETE after the above process. To examine the relationships between politically-themed stocks, we constructed politically-themed stock networks based on causal relationships using entropy-based approaches. Using the constructed politically-themed stock networks, we validate the two characteristics as we mentioned before based on the network measures and network dynamics. 

The contributions of this study can be summarized as three aspects. First, most prior research determined the politically-themed stocks from the occurrence of positive CAR during the political event periods. However, these results are unclear as to whether the occurrence of abnormal returns is influenced by politicians’ actions. Additionally, the stocks designated as politically-themed stocks in those studies were subjectively set by the researchers. However, the affected stocks were politically-themed and found that some stocks had known politically-themed stocks based on the definition of politically-themed stocks. Second, we verified the leading stocks from unequal values of the network analysis results that ETE of politically-themed stock networks show. Finally, to validate the positive CAR effect of prior research, we analyzed the network dynamics around political events. From the results, we found the trends of increasing ETE before and after political events. This clear trend means that the politically-themed stocks’ price movement trends its change around politically-themed stocks. Based on the insight from the network dynamics, we built investment portfolios using the PSI, and as a result, we found that the investment portfolio consists of politically-themed stocks using the PSI that can benchmark the index during the election period. These results can verify the CAR-related results of prior studies and the existence of eccentric movements and changes before and after political events simultaneously. The last two points can be evidence of Korean stock markets’ instability, similar to previous studies of politically-themed stocks in Korea.

We have three points that summarize this study’s limitations. First, we limited the factors affecting the abnormal returns of stocks only to the PSI. However, abnormal returns of stocks are the combined results of numerous factors in the real world. The second limitation is that the computed PSI can be different because the PSI values depend on the sentiment analysis results, which can be changed by the text data, machine-learning models, and hyperparameters. We adopted bidirectional LSTM as a machine-learning model for sentiment analysis because LSTM is one of the most commonly used methods in the recent 5 years in sentiment analysis based on the Korean language. However, well-performed machine-learning models like the transformer [61], BERT [62], and hybrid model [63] are released nowadays. For example, if those models are used for conducting sentiment analysis, it is believed that they may produce better results to detect politically-themed stocks based on the methodology of this paper. Finally, the computation time for constructing politically-themed stock networks was not considered. If someone builds a larger politically-themed stock networks to verify the network dynamics in this study, computation time will be an essential factor to consider. Thus, it is necessary to analyze further factors affecting abnormal returns and optimize factors related to calculation time, considering the consistency of results to construct advanced types of politically-themed networks.

Consequently, we developed a novel method to select the politically-themed stocks of politicians quantitatively based on sentiment analysis and entropy-based information flow measures. Additionally, we empirically analyzed the main characteristics of politically-themed stocks and developed politically-themed stock networks focusing on their causal relationships. Besides, this study’s contribution is that we came up with a new method to verify common knowledge of politically-themed stocks.

As a follow-up task, we will conduct studies to find other kinds of thematic stocks that can be analyzed from this perspective and develop novel methods for detecting non-thematic stocks’ relationships based on information theory and behavioral finance.

## Figures and Tables

**Table 1:** Main political events in Korea during the research period.

Date	Description
13 April 2016	Twentieth Legislative Election
10 March 2017	Impeachment of the Eighteenth President
9 May 2017	Nineteenth Presidential Election
13 June 2018	Seventh Local Election
15 August 2020	Twenty-First Legislative Election

**Table 2:** Brief information of 12 selected politicians.

Politician	Political Orientation	Presidential Elected Experience	Presidential Election Experience	Election (Presidential, Legislative, and Local) Experience	Strongly Mentioned as a Presidential Candidate from the Press
A	Right-Wing	O	O	O	O
B	Right-Wing	X	O	O	O
C	Right-Wing	X	X	O	O
D	Right-Wing	X	X	O	O
E	Right-Wing	X	O	O	O
F	Right-Wing	X	O	O	O
G	Neutral	X	X	X	O
H	Left-Wing	X	X	X	O
I	Left-Wing	X	X	O	O
J	Left-Wing	X	O	O	O
K	Left-Wing	X	X	O	O
L	Left-Wing	O	O	O	O

**Table 3:** Descriptive statistics in accordance with the rate of change (ROC) of PSIs.

Politician	Mean (%)	Std. Dev (%)	Min (%)	Max (%)	Q1 (%)	Median (%)	Q3 (%)	Skewness	Kurtosis	W	JB	$Q^{2}\left(10\right)$	$DF_{\tau }$	LM
A	$1.06\times 10^{-9}$	1.060	−7.711	5.433	−0.568	−0.009	0.594	−0.273	5.673	0.949 ***	1474.051 ***	48.168 ***	−12.028 ***	8.451 **
B	$2.54\times 10^{-10}$	1.492	−8.593	6.614	−0.775	−0.088	0.666	0.271	3.103	0.954 ***	449.521 ***	11.023	−29.848 ***	18.647 ***
C	$4.71\times 10^{-10}$	1.130	−8.508	8.121	−0.590	−0.045	0.526	0.318	8.517	0.918 ***	3312.044 ***	66.881 ***	−20.114 ***	25.244 ***
D	$1.20\times 10^{-9}$	1.207	−12.540	6.456	−0.642	−0.013	0.603	−0.762	13.475	0.912 ***	8354.230 ***	9.883	−30.803 ***	5.092 *
E	$-5.22\times 10^{-10}$	1.963	−15.240	9.683	−0.921	0.064	0.946	−0.483	6.580	0.925 ***	2007.802 ***	35.672 ***	−21.668 ***	6.463 **
F	$-3.81\times 10^{-10}$	2.012	−16.188	11.173	−0.804	0.105	0.964	−0.939	10.883	0.872 ***	5540.561 ***	9.983 **	−20.770 ***	2.226
G	$-2.24\times 10^{-9}$	1.972	−26.775	10.837	−0.849	−0.122	0.787	−2.213	35.542	0.810 ***	58,303.427 ***	14.509 *	−16.300 ***	7.910 **
H	$1.17\times 10^{-9}$	1.070	−7.491	5.728	−0.618	−0.027	0.575	0.123	5.194	0.942 ***	1226.414 ***	4.064	−21.996 ***	47.254 ***
I	$-1.15\times 10^{-9}$	1.319	−6.112	9.368	−0.674	−0.035	0.579	0.024	3.942	0.965 ***	704.575 ***	39.647 ***	−21.273 ***	8.406 **
J	$-7.28\times 10^{-10}$	1.083	−6.514	5.441	−0.568	−0.036	0.521	0.761	7.508	0.913 ***	2664.724 ***	23.538 ***	−12.017 ***	55.659 ***
K	$-3.81\times 10^{-10}$	1.507	−12.600	8.581	−0.758	−0.006	0.649	0.018	4.254	0.951 ***	820.630 ***	18.734 ***	−21.264 ***	0.829
L	$-9.54\times 10^{-10}$	0.151	−1.260	0.858	−0.076	−0.001	0.065	0.143	9.755	0.891 ***	4324.821 ***	16.220 *	−17.092 ***	6.481 **

Notes: W, JB, $Q^{2}\left(10\right),{\;\mathrm{DF}}_{\tau }$ and LM is the test statistic of the Shapiro–Wilk test and Jarque–Bera test for normality, the Ljung–Box test with 10-order serial autocorrelation in squared abnormal return data, the augmented Dickey–Fuller test, and White test each. ***, **, * means that the null hypothesis of normality, no autocorrelation in residuals, non-stationary, and homoscedasticity is rejected at the 10 percent, 5 percent, and 1 percent significance level each.

**Table 4:** Best-fit distribution in the rate of change (ROC) of PSIs.

Politician	Best-Fit Distribution
A	Noncentral t-distribution (ν=4.49, μ=0.10)
B	Johnson’s $S_{u}$ distribution (ε = −0.21, λ =1.20)
C	Noncentral t-distribution (ν=3.42, μ=0.27)
D	Lévy alpha-stable distribution (α=1.75, β=0.11)
E	Johnson’s $S_{u}$ distribution (ε=0.09, λ=1.13)
F	Lévy alpha-stable distribution (α=1.54, β=−0.24)
G	Noncentral t-distribution (ν=0.33, μ=−0.45)
H	Noncentral t-distribution (ν=3.78, μ=0.29)
I	Noncentral t-distribution (ν=4.70, μ=0.30)
J	Noncentral t-distribution (ν=3.17, μ=0.21)
K	Noncentral t-distribution (ν=3.57, μ=0.24)
L	t-distribution (ν=2.88)

**Table 5:** The number and proportion of stocks that have ETE-based causal relationships from ROC of PSIs to abnormal returns.

Significance Level α	$H_{0}\;$ Rejected (TE≠0)	$H_{0}\;\mathrm{Accepted}$
0.01	177 (93.65%)	12 (6.35%)
0.05	183 (96.83%)	6 (3.17%)
0.1	184 (97.35%)	5 (2.65%)

**Table 6:** Politician H’s example of measuring rank correlations between de-identified numbers and ETE rank values in descending order.

Politically-Themed Stock	Politician-Related Search Volume	Rank	ETE Values	Rank
H-1	93,800	1	0.1266	1
H-2	29,600	2	0.1024	6
H-3	11,000	3	0.0506	9
H-4	8660	4	0.1112	5
H-5	8420	5	0.1251	2
H-6	6410	6	0.1115	4
H-7	5350	7	0.1157	3
H-8	3560	8	0.0799	8
H-9	2740	9	0.0872	7
H-10	2280	10	0.0331	10

**Table 7:** The number and proportion of stocks that have ETE-based causal relationships between politically-themed stocks in the same network.

Significance Level α	$H_{0}\;$ Rejected (TE≠0)	$H_{0}\;\mathrm{Accepted}$
0.01	1716 (79.81%)	434 (20.19%)
0.05	1741 (80.98%)	409 (19.02%)
0.1	1760 (81.86%)	390 (18.14%)

**Table 8:** The average rank correlation between node-level attributes and de-identified numbers of politically-themed stocks.

Politically-Themed Stock Networks	Leading Stocks				Following Stocks				
A	A-5	A-7	A-16		A-2	A-5	A-7	A-16	
B	B-3	B-4	B-5	B-6	B-4	B-5	B-7		
C	C-4	C-7	C-9	C-20	C-4	C-9	C-12	C-20	
D	D-1	D-14			D-1	D-9	D-14		
E	E-1	E-2	E-4	E-10	E-1	E-8	E-9		
F	F-4	F-13			F-13	F-14			
G	G-1	G-4	G-8		G-1	G-3	G-4	G-6	G-8
H	H-2	H-4			H-2	H-4	H-7	H-8	
I	I-2	I-8	I-9		I-1	I-2	I-8	I-9	
J	J-1	J-2	J-13	J-20	J-1	J-4	J-20		
K	K-8	K-9	K-20		K-9	K-11	K-20		
L	L-1	L-6	L-12		L-6	L-8	L-11	L-12	

**Table 9:** Points in time for conducting network dynamics analysis before and after political events.

Date	Name	Description
13 April 2016	Period 1	Twentieth Legislative Election
10 March 2017	Period 2	Impeachment of the Eighteenth President
9 May 2017	Period 3	Nineteenth Presidential Election
13 June 2018	Period 4	Seventh Local Election
17 April 2019	Period 5	Control Point in Time
15 April 2020	Period 6	Twenty-First Legislative Election

## Data Availability

Data available on request due to restrictions eg privacy or ethical. The data presented in this study are available on request from the corresponding author. The data are not publicly available due to political contents in this paper.

## Appendix A

**Table A1:** Descriptive statistics of abnormal return data.

Candidates	Mean (%)	Std. Dev (%)	Min (%)	Max (%)	Q1 (%)	Median (%)	Q3 (%)	Skewness	Kurtosis	W	JB	$Q^{2}\left(10\right)$	$DF_{\tau }$	LM
A-01	7.80$\times 10^{-12}$	3.470	−20.787	27.004	−1.421	−0.273	1.031	2.301	18.312	0.772 ***	16,204.559 ***	13.219 **	−25.063 ***	3.967
A-02	−5.55$\times 10^{-12}$	2.668	−17.453	29.779	−1.217	−0.156	1.002	1.875	20.710	0.835 ***	20,131.236 ***	52.659 ***	−34.344 ***	15.700 ***
A-03	3.30$\times 10^{-12}$	3.208	−14.180	28.970	−1.406	−0.149	1.084	2.598	21.959	0.795 ***	23,144.340 ***	5.249	−13.742 ***	9.822 ***
A-04	−5.91$\times 10^{-12}$	3.817	−32.798	28.843	−1.902	−0.182	1.383	1.256	16.455	0.827 ***	12,590.051 ***	11.554	−32.275 ***	4.553
A-05	4.11$\times 10^{-12}$	3.377	−15.031	27.071	−1.750	−0.194	1.313	2.413	16.083	0.831 ***	12,816.687 ***	15.880 **	−33.985 ***	23.307 ***
A-06	5.22$\times 10^{-12}$	2.486	−8.714	25.474	−1.335	−0.197	1.032	2.101	15.794	0.872 ***	12,139.403 ***	6.000	−12.102 ***	1.323
A-07	−3.69$\times 10^{-12}$	3.814	−26.225	28.560	−1.365	−0.226	0.996	1.872	16.134	0.759 ***	12,467.613 ***	67.972 ***	−13.163 ***	31.027 ***
A-08	1.49$\times 10^{-11}$	3.594	−24.164	27.991	−1.474	−0.151	1.063	0.845	15.656	0.786 ***	11,266.064 ***	49.046 ***	−19.706 ***	46.191 ***
A-09	−1.00$\times 10^{-11}$	3.766	−18.430	25.701	−1.953	−0.105	1.621	0.396	4.393	0.944 ***	903.625 ***	2.967	−30.040 ***	30.401 ***
A-10	−2.19$\times 10^{-12}$	2.330	−13.054	21.419	−1.318	−0.144	1.054	1.409	12.899	0.900 ***	7921.117 ***	51.823 ***	−36.934 ***	6.551 **
A-11	−3.53$\times 10^{-12}$	2.138	−19.056	17.697	−1.188	−0.137	1.015	0.419	11.996	0.910 ***	6567.775 ***	4.942	−25.476 ***	3.975
A-12	−1.15$\times 10^{-11}$	2.377	−12.246	12.276	−1.441	−0.215	1.220	0.467	3.177	0.959 ***	497.240 ***	4.094	−24.952 ***	0.539
A-13	−1.11$\times 10^{-11}$	3.726	−25.055	25.375	−1.469	−0.174	1.306	0.161	12.625	0.827 ***	7244.397 ***	12.892 ***	−11.937 ***	31.798 ***
A-14	−5.19$\times 10^{-12}$	2.454	−12.040	12.829	−1.485	−0.085	1.235	0.518	2.701	0.967 ***	379.422 ***	12.154**	−34.375 ***	3.761
A-15	−9.62$\times 10^{-12}$	1.965	−10.178	25.993	−0.861	−0.095	0.746	3.785	45.030	0.749 ***	94,777.412 ***	43.098 ***	−36.279 ***	11.138 ***
A-16	−1.18$\times 10^{-12}$	3.831	−17.361	27.185	−1.807	−0.243	1.138	2.207	13.521	0.805 ***	9194.960 ***	20.524 ***	−15.659 ***	21.140 ***
B-01	1.08$\times 10^{-11}$	3.373	−28.367	25.368	−1.212	−0.240	0.830	2.206	21.515	0.739 ***	21,922.672 ***	2.403	−17.812 ***	19.946 ***
B-02	−1.41$\times 10^{-12}$	5.543	−35.591	26.231	−2.070	−0.064	1.784	0.190	10.808	0.818 ***	5311.843 ***	31.042 ***	−16.449 ***	24.122 ***
B-03	1.52$\times 10^{-12}$	4.210	−36.071	25.843	−1.835	−0.112	1.572	−0.080	12.515	0.859 ***	7115.398 ***	21.088 ***	−17.160 ***	17.411 ***
B-04	−1.07$\times 10^{-11}$	2.185	−9.647	24.107	−0.959	−0.059	0.815	1.799	19.747	0.837 ***	18,308.790 ***	0.979	−13.356 ***	3.399
B-05	−3.53$\times 10^{-12}$	3.701	−18.392	25.243	−1.670	−0.217	1.482	1.167	10.089	0.869 ***	4871.829 ***	12.785 ***	−32.350 ***	28.507 ***
B-06	3.97$\times 10^{-12}$	2.552	−30.978	19.421	−1.021	−0.069	1.010	−0.908	28.046	0.811 ***	35,893.676 ***	8.621	−33.934 ***	15.433 ***
B-07	7.18$\times 10^{-12}$	2.976	−12.206	26.534	−1.613	−0.043	1.270	1.896	14.062	0.879 ***	9639.999 ***	24.486 ***	−24.191 ***	20.188 ***
B-08	−1.49$\times 10^{-11}$	3.237	−30.703	25.917	−1.364	−0.184	1.229	0.280	16.671	0.836 ***	12,640.621 ***	5.655	−34.332 ***	7.485 **
B-09	−8.64$\times 10^{-12}$	2.729	−15.923	25.605	−1.200	−0.130	1.025	1.443	16.376	0.851 ***	12,563.078 ***	9.203 **	−11.495 ***	24.084 ***
B-10	−9.82$\times 10^{-12}$	1.616	−7.974	15.490	−0.711	−0.046	0.692	1.176	11.936	0.893 ***	6723.759 ***	47.183 ***	−22.687 ***	24.704 ***
C-01	9.53$\times 10^{-12}$	3.111	−20.753	26.729	−1.127	−0.125	0.870	1.925	20.795	0.761 ***	20,325.327 ***	57.728 ***	−14.387 ***	14.925 ***
C-02	3.82$\times 10^{-12}$	3.396	−17.552	27.474	−1.272	−0.168	1.015	2.582	23.560	0.727 ***	26,442.857 ***	31.807 ***	−10.705 ***	3.519
C-03	−1.58$\times 10^{-12}$	2.703	−16.440	26.031	−1.083	−0.208	0.853	2.713	28.390	0.752 ***	37,974.463 ***	2.433	−27.099 ***	64.061 ***
C-04	−9.94$\times 10^{-12}$	1.565	−10.132	18.293	−0.623	0.038	0.626	2.006	30.090	0.780 ***	41,880.919 ***	85.083 ***	−10.370 ***	13.817 ***
C-05	1.42$\times 10^{-12}$	4.138	−22.418	28.033	−1.913	−0.125	1.436	1.348	10.559	0.855 ***	5395.664 ***	8.653	−30.719 ***	0.704
C-06	1.78$\times 10^{-12}$	2.710	−21.275	16.383	−1.330	−0.090	1.174	−0.321	10.253	0.893 ***	4792.655 ***	44.069 ***	−19.982 ***	6.696**
C-07	6.14$\times 10^{-12}$	2.254	−9.081	22.361	−0.945	−0.049	0.830	2.086	21.003	0.828 ***	20,839.728 ***	11.567 *	−9.865 ***	16.537 ***
C-08	8.21$\times 10^{-12}$	3.331	−16.212	27.745	−1.487	−0.115	1.069	2.032	15.731	0.823 ***	11,998.067 ***	3.649	−34.585 ***	6.697**
C-09	9.54$\times 10^{-12}$	3.450	−16.718	27.182	−1.375	−0.149	1.190	1.466	13.311	0.844 ***	8440.814 ***	36.586 ***	−20.372 ***	26.299 ***
C-10	−1.78$\times 10^{-12}$	3.349	−34.865	26.626	−1.305	−0.128	1.051	0.145	22.116	0.788 ***	22,227.992 ***	11.849	−21.223 ***	5.093 *
C-11	−7.95$\times 10^{-12}$	4.274	−35.237	27.190	−1.836	−0.273	1.454	1.205	15.230	0.808 ***	10,802.502 ***	4.836	−13.864 ***	8.709 **
C-12	1.65$\times 10^{-12}$	3.362	−23.065	26.314	−1.152	−0.111	0.908	2.267	24.303	0.696 ***	27,778.975 ***	91.639 ***	−12.001 ***	33.674 ***
C-13	7.6$5\times 10^{-13}$	3.217	−34.749	27.026	−1.437	−0.128	1.201	−0.102	20.269	0.840 ***	18,668.992 ***	134.787 ***	−17.429 ***	9.457 ***
C-14	−6.16$\times 10^{-12}$	1.830	−9.118	11.841	−0.927	−0.092	0.749	1.093	7.310	0.898 ***	2643.939 ***	18.650 ***	−31.728 ***	0.865
C-15	2.30$\times 10^{-11}$	3.111	−14.229	28.364	−1.321	−0.163	1.096	3.144	25.279	0.754 ***	30,845.634 ***	7.469	−11.609 ***	2.673
C-16	−1.54$\times 10^{-11}$	1.600	−14.272	12.428	−0.688	−0.078	0.630	0.439	13.711	0.854 ***	8574.415 ***	12.170 *	−37.920 ***	13.444 ***
C-17	6.90$\times 10^{-12}$	3.112	−14.170	27.238	−1.406	−0.130	1.112	2.222	19.833	0.825 ***	18,774.823 ***	3.609	−21.055 ***	2.559
C-18	2.12$\times 10^{-11}$	3.330	−16.444	26.193	−1.591	−0.148	1.550	0.819	9.064	0.902 ***	3852.926 ***	7.347	−24.656 ***	5.048*
C-19	6.51$\times 10^{-12}$	1.512	−10.015	6.066	−0.785	0.027	0.763	−0.216	3.445	0.962 ***	546.366 ***	10.864*	−32.629 ***	11.131 ***
C-20	4.17$\times 10^{-12}$	3.031	−12.961	26.511	−1.345	−0.113	0.977	2.800	22.170	0.775 ***	23,767.101 ***	6.139	−35.932 ***	8.532 **
D-01	2.03$\times 10^{-12}$	3.901	−37.401	26.118	−1.394	−0.208	1.009	0.130	19.133	0.774 ***	16,634.326 ***	9.221	−21.317 ***	9.172 **
D-02	−6.99$\times 10^{-11}$	2.891	−36.989	26.760	−1.251	−0.143	1.002	−0.355	39.303	0.772 ***	70,225.141 ***	44.244 ***	−10.123 ***	8.122 **
D-03	−1.09$\times 10^{-11}$	1.097	−6.594	6.330	−0.519	−0.008	0.474	0.116	6.644	0.904 ***	2006.068 ***	14.626**	−13.331 ***	22.505 ***
D-04	−3.75$\times 10^{-11}$	4.474	−35.037	27.012	−2.038	−0.280	1.553	0.801	11.991	0.838 ***	6647.420 ***	6.717	−33.319 ***	2.870
D-05	−1.27$\times 10^{-12}$	3.225	−30.377	26.179	−1.269	−0.117	1.032	1.017	20.424	0.804 ***	19,142.688 ***	6.960	−24.761 ***	28.871 ***
D-06	5.28$\times 10^{-12}$	2.679	−11.842	26.184	−1.061	−0.083	0.924	1.672	14.589	0.855 ***	10,179.765 ***	56.458 ***	−26.558 ***	36.793 ***
D-07	−2.26$\times 10^{-11}$	2.777	−30.993	26.258	−0.990	−0.144	0.677	0.286	29.566	0.769 ***	39,738.706 ***	8.056	−33.922 ***	21.451 ***
D-08	5.50$\times 10^{-12}$	2.465	−14.390	26.132	−1.166	−0.067	0.913	1.848	17.038	0.854 ***	13,812.732 ***	8.070	−33.352 ***	1.314
D-09	−2.05$\times 10^{-12}$	4.213	−29.333	25.244	−1.834	−0.220	1.488	0.481	10.900	0.847 ***	5438.065 ***	5.556	−26.558 ***	11.502 ***
D-10	3.01$\times 10^{-12}$	2.365	−9.081	24.074	−1.211	−0.186	0.880	2.568	18.818	0.834 ***	17,294.882 ***	86.078 ***	−12.209 ***	33.626 ***
D-11	−2.17$\times 10^{-11}$	3.120	−18.519	29.473	−1.620	−0.143	1.509	1.400	12.646	0.895 ***	7622.858 ***	7.337	−35.180 ***	7.373 **
D-12	3.81$\times 10^{-12}$	3.833	−14.891	26.985	−1.845	−0.459	1.272	2.800	16.140	0.782 ***	13,269.830 ***	6.964	−11.839 ***	9.565 ***
D-13	−1.81$\times 10^{-12}$	3.184	−31.321	26.255	−1.663	−0.165	1.343	0.845	19.442	0.853 ***	17,303.858 ***	2.330	−17.525 ***	13.720 ***
D-14	7.01$\times 10^{-12}$	3.538	−32.955	27.001	−1.521	−0.118	1.243	0.874	18.598	0.811 ***	15,854.178 ***	11.293	−12.440 ***	2.636
D-15	−5.58$\times 10^{-11}$	3.640	−19.020	27.464	−1.480	−0.265	1.092	2.311	16.977	0.784 ***	14,071.233 ***	4.346	−13.191 ***	1.681
D-16	7.74$\times 10^{-12}$	3.668	−12.792	26.526	−1.895	−0.314	1.353	1.511	8.788	0.889 ***	3923.967 ***	7.059	−18.138 ***	1.728
D-17	−8.38$\times 10^{-12}$	1.766	−12.504	11.619	−0.940	−0.130	0.770	0.490	6.122	0.924 ***	1744.632 ***	7.394	−18.965 ***	4.804*
D-18	1.31$\times 10^{-10}$	1.014	−6.711	6.103	−0.491	0.036	0.514	−0.349	4.396	0.952 ***	898.522 ***	20.885 ***	−35.349 ***	0.286
E-01	2.88$\times 10^{-12}$	3.805	−14.599	26.297	−1.897	−0.008	1.614	1.022	6.172	0.922 ***	1920.204 ***	6.248	−12.061 ***	28.562 ***
E-02	6.45$\times 10^{-12}$	2.328	−21.137	12.143	−1.351	0.005	1.162	−0.407	7.558	0.948 ***	2623.226 ***	22.451 ***	−33.628 ***	4.229
E-03	3.82$\times 10^{-12}$	4.092	−26.616	26.400	−1.575	−0.030	1.268	1.596	16.037	0.762 ***	12,150.187 ***	13.478**	−13.065 ***	4.177
E-04	9.79$\times 10^{-12}$	2.890	−19.247	26.220	−1.130	0.040	1.045	0.412	10.907	0.887 ***	5433.862 ***	17.184**	−12.861 ***	33.226 ***
E-05	−1.79$\times 10^{-11}$	3.990	−22.241	26.109	−1.973	−0.116	1.693	0.929	10.238	0.876 ***	4917.448 ***	14.335**	−33.884 ***	17.797 ***
E-06	−2.34$\times 10^{-12}$	4.244	−29.222	26.417	−1.901	−0.121	1.489	1.364	11.891	0.830 ***	6762.581 ***	24.438 ***	−12.076 ***	36.809 ***
E-07	−7.41$\times 10^{-12}$	3.723	−15.106	26.450	−1.892	−0.316	1.394	1.854	11.657	0.854 ***	6800.392 ***	1.242	−19.430 ***	5.024 *
E-08	−1.20$\times 10^{-12}$	4.189	−25.016	26.192	−1.729	−0.028	1.492	0.965	10.760	0.850 ***	5428.559 ***	22.024 ***	−13.620 ***	8.967 **
E-09	−3.84$\times 10^{-12}$	3.528	−19.795	26.264	−1.304	0.041	1.085	1.670	15.070	0.818 ***	10,826.834 ***	7.994	−17.424 ***	6.753**
E-10	−4.65$\times 10^{-12}$	1.894	−12.502	15.700	−0.762	0.045	0.786	0.136	11.172	0.870 ***	5672.015 ***	47.019 ***	−31.348 ***	39.182 ***
F-01	3.06$\times 10^{-12}$	3.220	−29.617	22.745	−1.257	0.012	1.225	0.129	15.894	0.819 ***	11,478.698 ***	18.111 ***	−11.725 ***	1.031
F-02	−8.09$\times 10^{-12}$	4.189	−25.823	26.359	−1.741	−0.005	1.673	0.674	11.929	0.835 ***	6546.291 ***	15.344 ***	−32.680 ***	0.415
F-03	1.33$\times 10^{-11}$	3.672	−18.643	26.242	−1.813	−0.094	1.810	0.688	6.642	0.926 ***	2088.889 ***	9.554	−33.704 ***	6.008 **
F-04	−1.27$\times 10^{-11}$	3.189	−21.000	26.278	−1.304	−0.038	1.105	1.034	14.475	0.835 ***	9713.357 ***	31.986 ***	−15.385 ***	42.358 ***
F-05	2.10$\times 10^{-12}$	3.218	−16.666	26.457	−1.429	−0.062	1.181	1.780	16.560	0.831 ***	13,037.781 ***	33.974 ***	−12.295 ***	9.246 ***
F-06	3.77$\times 10^{-11}$	4.197	−23.820	26.333	−1.862	−0.027	1.673	0.644	9.085	0.880 ***	3823.764 ***	12.930*	−33.231 ***	0.531
F-07	6.28$\times 10^{-12}$	3.608	−23.129	26.241	−1.339	−0.062	1.280	1.757	18.924	0.787 ***	16,835.769 ***	11.497 ***	−17.698 ***	3.663
F-08	−1.31$\times 10^{-11}$	4.064	−35.929	26.120	−1.824	−0.069	1.545	0.655	14.288	0.840 ***	9351.655 ***	32.411 ***	−31.781 ***	4.442
F-09	−1.17$\times 10^{-11}$	3.623	−24.113	26.179	−1.527	−0.041	1.325	0.750	12.540	0.842 ***	7245.007 ***	3.477	−36.828 ***	5.378*
F-10	6.89$\times 10^{-12}$	3.841	−35.558	26.224	−1.412	−0.017	1.148	1.048	21.981	0.755 ***	22,155.445 ***	27.797 ***	−20.531 ***	16.403 ***
F-11	2.23$\times 10^{-12}$	3.370	−20.350	26.290	−1.481	0.041	1.423	0.932	12.630	0.874 ***	7404.776 ***	22.033 ***	−31.001 ***	3.784
F-12	1.05$\times 10^{-11}$	2.629	−16.112	25.808	−1.249	0.051	1.145	1.268	15.872	0.868 ***	11,738.897 ***	1.687	−33.625 ***	2.562
F-13	4.94	3.716	−15.041	26.191	−1.493	−0.072	1.376	1.526	11.640	0.848 ***	6579.876 ***	36.047 ***	−14.147 ***	26.591 ***
F-14	−2.50$\times 10^{-12}$	3.114	−26.721	21.150	−1.315	−0.001	1.257	−0.429	16.066	0.841 ***	11,759.776 ***	52.564 ***	−16.396 ***	17.000 ***
F-15	7.44$\times 10^{-12}$	4.421	−34.481	26.310	−1.954	−0.070	1.692	1.049	12.675	0.839 ***	7498.967 ***	2.483	−31.225 ***	1.604
F-16	1.16$\times 10^{-12}$	3.067	−26.841	26.340	−1.137	0.079	0.866	1.477	28.730	0.729 ***	37,908.277 ***	17.600 ***	−11.476 ***	4.082
F-17	−1.36$\times 10^{-11}$	2.929	−15.343	19.410	−1.316	−0.075	1.257	0.340	6.477	0.911 ***	1924.913 ***	14.506 **	−30.979 ***	26.173 ***
G-01	8.48$\times 10^{-12}$	4.353	−34.607	27.240	−1.896	−0.319	1.457	0.820	15.208	0.815 ***	10628.920 ***	3.686	−31.566 ***	13.029 ***
G-02	6.45$\times 10^{-12}$	3.132	−29.513	24.885	−1.254	−0.268	1.049	0.669	23.551	0.772 ***	25,283.544 ***	11.570 **	−12.120 ***	7.194 **
G-03	−2.13$\times 10^{-12}$	4.173	−34.452	27.415	−1.635	−0.198	1.155	0.755	17.326	0.782 ***	13,741.711 ***	20.899 ***	−12.797 ***	27.935 ***
G-04	4.83$\times 10^{-12}$	4.379	−35.463	26.563	−2.037	−0.266	1.642	0.178	13.214	0.845 ***	7937.386 ***	131.945 ***	−14.463 ***	23.138 ***
G-05	6.85$\times 10^{-12}$	4.221	−34.993	26.490	−1.836	−0.181	1.549	0.492	13.293	0.841 ***	8070.649 ***	11.632	−33.966 ***	4.820 *
G-06	−1.32$\times 10^{-11}$	3.682	−29.240	26.548	−1.756	−0.181	1.221	1.980	19.077	0.777 ***	17,251.824 ***	10.754	−14.896 ***	5.412 *
G-07	6.80$\times 10^{-12}$	3.513	−16.616	26.643	−1.494	−0.175	1.232	1.939	14.102	0.830 ***	9721.093 ***	7.208	−15.522 ***	4.083
G-08	3.86$\times 10^{-13}$	4.717	−35.805	28.496	−1.766	−0.276	1.336	0.424	18.071	0.741 ***	14,869.606 ***	66.045 ***	−13.646 ***	19.922 ***
G-09	7.85$\times 10^{-12}$	3.732	−38.082	28.307	−1.725	−0.215	1.196	0.141	21.983	0.788 ***	21,962.016 ***	49.376 ***	−9.753 ***	10.230 ***
G-10	−5.95$\times 10^{-12}$	3.800	−33.613	27.678	−1.642	−0.189	1.370	0.626	15.746	0.829 ***	11,335.453 ***	29.833 ***	−14.901 ***	5.778 *
G-11	2.12$\times 10^{-11}$	2.239	−18.530	25.439	−0.876	−0.092	0.636	2.737	34.196	0.723 ***	54,514.188 ***	61.706 ***	−30.283 ***	46.037 ***
G-12	7.71$\times 10^{-12}$	1.973	−11.736	21.136	−0.887	−0.097	0.740	1.867	20.468	0.839 ***	19,673.065 ***	9.448 *	−19.788 ***	1.735
G-13	8.42$\times 10^{-12}$	2.161	−12.719	20.362	−0.917	−0.070	0.718	2.331	22.986	0.806 ***	25,002.236 ***	38.430 ***	−33.532 ***	45.435 ***
G-14	4.60$\times 10^{-12}$	3.175	−19.835	28.252	−1.501	−0.090	1.157	0.978	14.133	0.866 ***	9247.918 ***	6.620	−19.644 ***	3.660
G-15	−1.32$\times 10^{-12}$	2.485	−17.074	25.880	−1.090	−0.112	0.874	1.065	18.395	0.827 ***	15,581.134 ***	4.839	−12.711 ***	0.441
H-01	9.89$\times 10^{-12}$	3.129	−17.551	26.404	−1.415	−0.213	1.024	2.086	14.838	0.823 ***	10,797.339 ***	19.420 **	−31.925 ***	13.076 ***
H-02	1.95$\times 10^{-11}$	3.527	−36.557	27.629	−0.889	−0.068	0.732	1.216	30.366	0.655 ***	42,174.908 ***	14.998 **	−13.175 ***	41.835 ***
H-03	−1.64$\times 10^{-11}$	2.598	−14.843	25.573	−1.260	−0.144	0.978	2.592	26.724	0.794 ***	33,681.340 ***	73.141 ***	−17.645 ***	39.120 ***
H-04	−2.58$\times 10^{-12}$	2.966	−13.915	26.115	−1.144	−0.109	0.923	2.468	19.537	0.779 ***	18,455.896 ***	13.160	−25.689 ***	13.666 ***
H-05	−8.81$\times 10^{-12}$	4.605	−35.224	26.679	−1.984	−0.243	1.719	0.170	12.689	0.841 ***	7318.666 ***	6.126	−32.865 ***	0.080
H-06	2.98$\times 10^{-12}$	3.852	−14.686	26.599	−1.959	−0.249	1.587	1.130	6.756	0.917 ***	2305.123 ***	32.235 ***	−11.669 ***	5.109 *
H-07	−1.06$\times 10^{-11}$	4.302	−29.142	27.798	−1.463	−0.155	1.019	1.700	16.010	0.751 ***	12,172.832 ***	20.779 ***	−11.012 ***	6.447 **
H-08	7.18$\times 10^{-12}$	2.656	−13.958	26.870	−1.220	−0.121	1.037	2.216	24.134	0.829 ***	27,364.080 ***	3.773	−16.275 ***	0.593
H-09	−2.33	2.239	−20.620	21.711	−0.886	0.008	0.748	0.816	20.535	0.809 ***	19,281.080 ***	54.392 ***	−26.012 ***	11.528 ***
H-10	7.84$\times 10^{-12}$	2.580	−14.548	22.657	−1.259	−0.094	1.214	0.994	10.212	0.909 ***	4916.211 ***	4.808	−18.166 ***	21.983 ***
I-01	−1.35$\times 10^{-11}$	2.803	−18.463	25.475	−1.167	−0.185	0.953	1.789	19.678	0.802 ***	18,179.682 ***	131.776 ***	−18.146 ***	15.403 ***
I-02	−2.14$\times 10^{-12}$	2.280	−12.802	12.771	−1.201	−0.182	0.974	0.635	4.674	0.930 ***	1064.612 ***	7.036	−13.641 ***	32.277 ***
I-03	1.02$\times 10^{-10}$	4.324	−38.177	28.147	−1.813	−0.259	1.211	0.912	17.259	0.783 ***	13,684.372 ***	14.151	−32.771 ***	8.985**
I-04	−5.18$\times 10^{-12}$	3.041	−14.596	25.183	−1.468	−0.160	1.157	1.765	13.891	0.863 ***	9334.891 ***	15.990**	−13.131 ***	4.625*
I-05	1.84$\times 10^{-11}$	2.899	−17.801	28.158	−1.500	−0.154	1.012	2.386	19.500	0.821 ***	18,317.492 ***	3.246	−18.929 ***	23.810 ***
I-06	−1.43$\times 10^{-11}$	3.065	−16.087	26.893	−1.510	−0.184	1.158	2.113	17.876	0.842 ***	15,335.228 ***	11.307	−38.014 ***	3.282
I-07	7.72$\times 10^{-12}$	2.976	−12.206	26.534	−1.613	−0.043	1.270	1.896	14.062	0.879 ***	9639.999 ***	24.486 ***	−24.191 ***	20.188 ***
I-08	9.74$\times 10^{-12}$	3.077	−17.430	29.011	−1.063	−0.116	0.743	3.748	35.119	0.671 ***	58621.573 ***	41.746 ***	−11.816 ***	27.538 ***
I-09	2.38$\times 10^{-12}$	3.229	−16.694	26.554	−1.674	−0.253	1.373	1.528	13.340	0.869 ***	8509.681 ***	19.996 ***	−17.668 ***	5.378*
I-10	4.52$\times 10^{-12}$	2.737	−11.565	27.410	−1.152	−0.115	0.895	3.572	31.317	0.747 ***	46,905.279 ***	3.151	−11.253 ***	6.225**
I-11	−1.88$\times 10^{-12}$	3.509	−18.669	27.277	−1.367	−0.182	0.835	2.809	21.357	0.731 ***	22,168.525 ***	41.987 ***	−14.288 ***	25.685 ***
I-12	−9.82	2.395	−14.019	16.462	−1.254	−0.064	1.081	0.603	7.228	0.913 ***	2437.803 ***	1.731	−35.143 ***	1.976
I-13	2.04$\times 10^{-12}$	2.587	−15.653	26.689	−1.234	−0.036	1.035	1.038	16.233	0.866 ***	12,168.214 ***	3.293	−11.551 ***	14.246 ***
I-14	4.90$\times 10^{-12}$	2.684	−16.577	25.964	−1.206	−0.032	1.059	1.483	15.920	0.840 ***	11,915.415 ***	0.483 ***	−16.632 ***	6.738 **
I-15	−2.90$\times 10^{-12}$	4.395	−29.781	30.821	−1.714	−0.269	1.154	2.039	15.686	0.761 ***	11,939.073 ***	13.690 ***	−11.495 ***	69.411 ***
I-16	2.72$\times 10^{-12}$	2.334	−11.065	15.154	−1.292	−0.146	0.926	1.097	5.347	0.927 ***	1517.554 ***	8.285	−32.841 ***	1.264
J-01	7.48$\times 10^{-12}$	3.647	−20.694	26.734	−1.722	−0.211	1.323	2.218	15.988	0.824 ***	12,512.961 ***	81.391 ***	−17.027 ***	13.867 ***
J-02	8.34$\times 10^{-12}$	2.865	−15.643	24.722	−1.097	−0.113	0.872	1.556	15.137	0.790 ***	10,851.742 ***	28.211 ***	−15.795 ***	57.420 ***
J-03	−1.26$\times 10^{-11}$	3.659	−35.366	26.660	−1.576	−0.217	1.129	1.335	20.589	0.784 ***	19,586.324 ***	19.488 ***	−21.345 ***	20.661 ***
J-04	−1.25$\times 10^{-11}$	3.785	−24.498	26.709	−1.676	−0.310	1.142	1.380	13.706	0.820 ***	8881.830 ***	32.269 ***	−20.390 ***	2.641
J-05	4.21 $\times 10^{-12}$	2.842	−30.144	25.488	−1.384	−0.122	1.238	−0.043	19.746	0.867 ***	17,716.233 ***	4.210	−35.345 ***	3.986
J-06	−7.12$\times 10^{-12}$	4.177	−33.530	27.162	−1.764	−0.277	1.526	0.980	13.960	0.829 ***	9027.748 ***	19.530 ***	−14.803 ***	15.601 ***
J-07	4.06$\times 10^{-13}$	2.723	−25.422	21.992	−1.401	−0.183	1.152	0.571	13.884	0.879 ***	8816.387 ***	40.553 ***	−22.695 ***	14.411 ***
J-08	5.75$\times 10^{-12}$	3.557	−22.377	26.068	−1.452	−0.116	1.246	1.067	14.715	0.823 ***	10,044.243 ***	35.216 ***	−15.326 ***	22.879 ***
J-09	−5.64$\times 10^{-12}$	2.630	−10.699	17.312	−1.426	−0.222	1.191	1.167	5.491	0.927 ***	1617.413 ***	11.337	−32.212 ***	0.764
J-10	−1.30$\times 10^{-12}$	3.735	−18.323	30.183	−1.632	−0.275	1.199	1.557	11.783	0.846 ***	6749.851 ***	9.396 *	−35.194 ***	14.362 ***
J-11	1.50$\times 10^{-11}$	2.671	−13.358	26.249	−1.361	−0.192	1.017	1.917	14.203	0.867 ***	9835.555 ***	3.845	−11.944 ***	9.406 ***
J-12	6.34$\times 10^{-12}$	3.274	−16.405	26.251	−1.660	−0.210	1.229	1.823	12.650	0.862 ***	7877.156 ***	27.135 ***	−33.929 ***	18.146 ***
J-13	−2.25$\times 10^{-11}$	2.837	−16.124	26.212	−1.196	−0.093	1.023	1.362	17.055	0.833 ***	13,554.021 ***	27.317 ***	−12.941 ***	27.029 ***
J-14	−4.40$\times 10^{-12}$	2.466	−16.903	26.446	−1.232	−0.119	1.151	1.482	19.050	0.872 ***	16,890.711 ***	10.216	−33.879 ***	0.636
J-15	−6.03$\times 10^{-12}$	3.754	−20.248	26.026	−1.914	−0.352	1.238	1.607	8.481	0.866 ***	3738.476 ***	7.931	−18.291 ***	1.735
J-16	−1.41$\times 10^{-11}$	3.856	−23.884	26.984	−1.726	−0.237	1.456	1.405	13.598	0.834 ***	8760.501 ***	11.731 *	−32.140 ***	3.139
J-17	−8.42$\times 10^{-12}$	3.057	−21.275	26.007	−1.608	−0.167	1.351	0.360	9.486	0.913 ***	4109.702 ***	2.502	−30.941 ***	0.534
J-18	−3.33$\times 10^{-12}$	3.386	−15.643	28.146	−1.689	−0.204	1.377	1.298	11.047	0.881 ***	5850.550 ***	9.023	−31.461 ***	7.991**
J-19	4.67$\times 10^{-12}$	3.289	−16.670	27.278	−1.688	−0.351	1.311	1.810	13.282	0.859 ***	8613.073 ***	21.878 ***	−12.093 ***	7.207**
J-20	−5.45$\times 10^{-12}$	3.120	−22.460	17.443	−1.317	−0.127	1.045	0.641	8.307	0.872 ***	3207.947 ***	59.533 ***	−13.868 ***	70.497 ***
J-21	−1.15$\times 10^{-12}$	2.023	−13.129	13.704	−1.013	−0.097	0.895	0.210	6.371	0.926 ***	1850.185 ***	27.896 ***	−20.997 ***	14.047 ***
K-01	−4.94$\times 10^{-12}$	3.658	−33.702	23.782	−1.578	−0.164	1.396	−0.371	15.419	0.848 ***	10,826.059 ***	6.526	−14.184 ***	0.496
K-02	1.27$\times 10^{-11}$	3.349	−14.596	26.030	−1.536	−0.210	1.292	2.425	18.411	0.819 ***	16,476.020 ***	3.060	−14.841 ***	2.954
K-03	4.19$\times 10^{-12}$	3.764	−27.868	28.539	−1.614	−0.201	1.252	2.162	19.281	0.778 ***	17,746.703 ***	10.383*	−16.646 ***	12.040 ***
K-04	2.33$\times 10^{-13}$	3.373	−25.693	27.896	−1.773	−0.288	1.314	1.050	12.386	0.886 ***	7169.811 ***	71.574 ***	−32.740 ***	6.017 **
K-05	−9.55$\times 10^{-12}$	3.483	−34.788	26.693	−1.441	−0.130	1.249	0.273	19.778	0.819 ***	17,786.721 ***	7.585	−36.677 ***	5.915 *
K-06	7.03$\times 10^{-12}$	3.033	−29.122	25.613	−1.413	−0.150	1.247	−0.019	16.239	0.861 ***	11,980.402 ***	25.279 ***	−19.633 ***	1.473
K-07	8.26$\times 10^{-11}$	2.349	−8.106	25.254	−1.141	−0.125	0.927	3.222	30.295	0.801 ***	43,605.779 ***	2.849	−18.967 ***	1.641
K-08	−9.03$\times 10^{-12}$	3.242	−15.056	26.815	−1.754	−0.190	1.401	1.792	11.862	0.873 ***	6978.277 ***	9.227	−13.369 ***	5.999 **
K-09	−8.01$\times 10^{-12}$	2.657	−16.187	25.194	−1.163	−0.191	0.996	2.352	25.198	0.778 ***	29,864.990 ***	10.380	−19.151 ***	18.640 ***
K-10	7.03$\times 10^{-12}$	3.183	−12.928	26.987	−1.336	−0.106	0.978	2.115	15.977	0.819 ***	12,414.995 ***	13.421 **	−34.775 ***	10.558 ***
K-11	2.93$\times 10^{-12}$	2.361	−14.868	26.247	−1.108	−0.046	0.940	1.551	20.038	0.857 ***	18,682.820 ***	4.013	−12.766 ***	2.856
K-12	2.69$\times 10^{-12}$	2.072	−13.266	11.806	−0.926	−0.046	0.880	0.156	6.546	0.918 ***	1949.383 ***	9.989 *	−13.625 ***	3.963
K-13	−5.98$\times 10^{-12}$	2.042	−11.094	16.164	−0.927	−0.006	0.759	1.176	11.361	0.864 ***	6115.036 ***	42.164 ***	−35.814 ***	2.079
K-14	7.96$\times 10^{-12}$	3.500	−29.094	28.686	−1.503	−0.167	1.051	1.613	19.497	0.777 ***	17,747.584 ***	7.036	−14.927 ***	0.555
K-15	3.45$\times 10^{-12}$	2.825	−11.172	26.385	−1.420	−0.226	1.083	2.336	16.530	0.837 ***	13,411.690 ***	11.081	−33.278 ***	1.568
K-16	8.16$\times 10^{-12}$	3.038	−16.617	25.885	−1.373	−0.162	1.016	2.535	21.352	0.780 ***	21,892.133 ***	10.306	−17.140 ***	0.813
K-17	−2.50$\times 10^{-12}$	4.159	−23.225	25.225	−1.982	−0.368	1.691	0.749	7.014	0.896 ***	2335.692 ***	36.769 ***	−16.713 ***	25.573 ***
K-18	8.46$\times 10^{-12}$	2.199	−8.483	26.292	−1.178	−0.149	0.862	2.583	23.235	0.845 ***	25,750.729 ***	11.590	−9.829 ***	3.241
K-19	−5.14$\times 10^{-12}$	3.512	−35.613	21.430	−1.363	−0.097	1.047	−1.397	25.052	0.788 ***	28,874.299 ***	109.555 ***	−10.360 ***	40.727 ***
K-20	1.39$\times 10^{-11}$	2.376	−14.444	19.772	−1.310	−0.038	1.139	0.943	9.326	0.919 ***	4112.398 ***	3.299	−21.947 ***	2.621
L-01	−1.29$\times 10^{-11}$	4.804	−34.134	29.581	−1.798	−0.253	1.268	1.562	13.355	0.765 ***	8548.411 ***	1.740	−13.269 ***	3.337
L-02	−6.79$\times 10^{-12}$	4.421	−30.571	27.679	−1.861	−0.213	1.204	1.575	13.293	0.793 ***	8479.967 ***	11.277	−31.998 ***	0.768
L-03	1.06$\times 10^{-11}$	2.486	−9.740	26.300	−1.280	−0.158	1.064	1.929	16.783	0.878 ***	13,477.710 ***	42.197 ***	−17.545 ***	11.435 ***
L-04	−8.62$\times 10^{-12}$	2.818	−26.526	26.572	−1.184	−0.118	1.133	−0.065	25.180	0.805 ***	28,811.198 ***	5.898	−22.251 ***	0.243
L-05	8.69$\times 10^{-13}$	3.214	−19.021	26.295	−1.569	−0.125	1.407	1.208	10.096	0.894 ***	4895.420 ***	154.856 ***	−11.770 ***	10.909 ***
L-06	−6.21$\times 10^{-12}$	4.030	−31.602	29.639	−1.654	−0.202	1.396	1.013	14.734	0.820 ***	10,049.810 ***	9.272	−34.450 ***	3.852
L-07	−2.82$\times 10^{-12}$	2.245	−10.034	11.762	−1.203	−0.165	1.065	0.491	2.463	0.965 ***	318.626 ***	9.282	−33.138 ***	3.543
L-08	2.03$\times 10^{-12}$	2.846	−11.274	26.496	−1.470	−0.162	1.092	1.435	9.721	0.908 ***	4668.026 ***	4.064	−19.677 ***	12.763 ***
L-09	−9.48$\times 10^{-12}$	4.679	−36.653	30.477	−2.192	−0.417	1.653	0.864	12.085	0.840 ***	6769.415 ***	2.035	−29.535 ***	1.952
L-10	−2.43$\times 10^{-12}$	2.147	−8.446	15.641	−1.163	−0.134	0.880	1.001	4.967	0.935 ***	1302.217 ***	7.029	−33.817 ***	12.282 ***
L-11	5.66$\times 10^{-12}$	3.597	−20.263	27.426	−1.556	−0.211	1.071	1.797	14.202	0.830 ***	9753.727 ***	6.046	−31.274 ***	5.357*
L-12	3.77$\times 10^{-13}$	1.693	−8.312	9.326	−0.989	−0.069	0.778	0.519	2.713	0.963 ***	382.672 ***	2.077 ***	−32.609 ***	30.995 ***
L-13	1.52$\times 10^{-11}$	2.379	−12.784	16.216	−1.378	−0.123	1.231	0.650	4.706	0.947 ***	1081.628 ***	8.179	−34.750 ***	9.345 ***
L-14	−6.24$\times 10^{-12}$	2.507	−12.918	22.254	−1.090	−0.048	0.944	1.263	13.868	0.856 ***	9027.632 ***	8.759	−17.650 ***	4.521
L-15	−1.10$\times 10^{-10}$	3.415	−26.605	26.900	−1.628	−0.141	1.308	1.038	16.555	0.839 ***	12,648.013 ***	0.853	−14.431 ***	4.752 *
L-16	5.88$\times 10^{-12}$	2.085	−8.557	13.335	−1.105	−0.057	0.914	0.624	4.288	0.946 ***	905.028 ***	3.536	−10.767 ***	1.844

Notes: W, JB, $Q^{2}\left(10\right),{\;\mathrm{DF}}_{\tau }$, and LM are the test statistics of the Shapiro–Wilk test and Jarque–Bera test for normality, the Ljung–Box test for 10-order serial autocorrelation in squared abnormal return data, the augmented Dickey–Fuller test, and the White test, respectively. ***, **, * means that the null hypothesis of normality, no autocorrelation in residuals, non-stationary, and homoscedasticity is rejected at the 10 percent, 5 percent, and 1 percent significance level, respectively.
